# Estrogens, age, and, neonatal stress: panic disorders and novel views on the contribution of non-medullary structures to respiratory control and CO_2_ responses

**DOI:** 10.3389/fphys.2023.1183933

**Published:** 2023-05-17

**Authors:** Richard Kinkead, Danuzia Ambrozio-Marques, Stéphanie Fournier, Marianne Gagnon, Loralie Mei Guay

**Affiliations:** Département de Pédiatrie, Research Center of the Québec Heart and Lung Institute, Université Laval, Quebec City, QC, Canada

**Keywords:** hyperventilation, sex-based differences, control of breathing, orexin, estradiol (17ß-estradiol), maternal separation anxiety

## Abstract

CO_2_ is a fundamental component of living matter. This chemical signal requires close monitoring to ensure proper match between metabolic production and elimination by lung ventilation. Besides ventilatory adjustments, CO_2_ can also trigger innate behavioral and physiological responses associated with fear and escape but the changes in brain CO_2_/pH required to induce ventilatory adjustments are generally lower than those evoking fear and escape. However, for patients suffering from panic disorder (PD), the thresholds for CO_2_-evoked hyperventilation, fear and escape are reduced and the magnitude of those reactions are excessive. To explain these clinical observations, Klein proposed *the false suffocation alarm hypothesis* which states that *many spontaneous panics occur when the brain’s suffocation monitor erroneously signals a lack of useful air, thereby maladaptively triggering an evolved suffocation alarm system*. After 30 years of basic and clinical research, it is now well established that anomalies in respiratory control (including the CO_2_ sensing system) are key to PD. Here, we explore how a stress-related affective disorder such as PD can disrupt respiratory control. We discuss rodent models of PD as the concepts emerging from this research has influenced our comprehension of the CO_2_ chemosensitivity network, especially structure that are not located in the medulla, and how factors such as stress and biological sex modulate its functionality. Thus, elucidating why hormonal fluctuations can lead to excessive responsiveness to CO_2_ offers a unique opportunity to gain insights into the neuroendocrine mechanisms regulating this key aspect of respiratory control and the pathophysiology of respiratory manifestations of PD.

## 1 Introduction and overview

As a chemist, Antoine Lavoisier was the first to acknowledge CO_2_ as a “fundamental component of living matter”. While his significant contributions to modern physiology did not save him from the guillotine, CO_2_ is now acknowledged as a chemical signal that requires close monitoring to ensure proper match between metabolic production and elimination by lung ventilation. Although accurate, this approach neglects the fact that CO_2_ accumulation is a sign of an impoverished air quality such that in many species (including rodents and humans), CO_2_ can trigger innate behavioral and physiological responses associated with fear and escape. These responses are highly adaptive because leaving the room (rather than hyperventilating) is perhaps the simplest (and most efficient) way to deal with a hypercarbic environment! Clearly, the changes in brain CO_2_/pH required to induce ventilatory adjustments are far lower than those evoking fear and escape (Guyenet and Bayliss, 2022), but in a subpopulation of patients suffering from anxiety disorders, the thresholds for CO_2_-evoked hyperventilation, fear and escape are reduced and the magnitude of those reactions are excessive. This trait can then initiate a vicious circle: if the increase in breathing is disproportionate, the perception of respiratory efforts along with the excessive CO_2_ loss (hypocapnia) can trigger a variety of physical sensations ranging from headaches to chest pain/discomfort (Gardner, 1996). Panic disorder (PD) patients misinterpret these physiological signals as life-threatening and experience strong emotional reactions that can lead to full-blown panic attacks encompassing fear of dying, shortness of breath, and a choking sensation (Gardner, 1996; Gorman et al., 2000; Nardi et al., 2009). To explain these clinical observations, Donald Klein proposed “*the false suffocation alarm hypothesis”* which states that “*many spontaneous panics occur when the brain’s suffocation monitor erroneously signals a lack of useful air, thereby maladaptively triggering an evolved suffocation alarm system*” (Klein, 1993). This model has been refined (Feinstein et al., 2022; Kinkead et al., 2022), but has generally stood the test of time. After 30 years of basic and clinical research, it is now well established that anomalies in respiratory control (including the CO_2_ sensing system) are key to PD. Moreover, the intense fear and anxiety experienced by PD patients highlight the functional and anatomical overlaps that exists between the neural circuits that control breathing and those that regulate emotions, fear and escape responses (Schenberg, 2016; Venkatraman et al., 2017). Each of these systems has been studied extensively in isolation, but in recent years, the study of their functional intersection has offered a novel and broader view of respiratory neurobiology. Recent work from Feldman and collaborators has deciphered the pathways by which inspiratory rhythm originating from the pre-Bötzinger complex affects emotions (Ashhad et al., 2022). Here, we will look at this relationship from a different angle; namely, we will explore how a stress-related affective disorder such as PD can influence respiratory control. We focus on rodent models of PD as the concepts emerging from this research has influenced our comprehension of the CO_2_ chemosensitivity network and how factors such as stress and biological sex modulate its functionality.

## 2 Panic disorder: Definitions and sex-based differences in respiratory manifestations of neural control dysfunction

Panic disorder is an anxiety disorder characterized by recurrent panic attacks that are acute, unexpected, and that occur without a clear trigger (World-Health-Organization, 1992; American-Psychiatric-Association, 1994). A panic attack is defined as an episode of overwhelming physical distress and cognitive anxiety during which the patient rapidly develops intense symptoms such as air hunger, sweating, heart palpitations, shortness of breath, hyperventilation, and fear of dying (Hoppe et al., 2012). As such, PD is perhaps one of the most overwhelming experiences that a person can endure (Moreira et al., 2013). While the DSM-V definition of PD spans across 13 different symptoms, the respiratory PD subtype has been identified as the most common, the most pervasive, and the most disabling form of PD (Roberson-Nay and Kendler, 2011). This demonstrates the prominence of the respiratory distress symptoms in PD (Wilhelm et al., 2001; Roberson-Nay et al., 2010; Hoppe et al., 2012; Rappaport et al., 2017) and, depending on theoretical standpoints, hyperventilation can thus be viewed as a cause, a correlate, or a consequence of panic attacks (Nardi et al., 2009).

During World War I, it was observed that CO_2_ rebreathing while wearing a gas mask can bring some soldiers to remove the gas mask and/or induce a panic attack (Ritchie, 1992); it was proposed at the time that soldiers prone to the “irritable heart” are excessively sensitive to this stimulus (Drury, 1918). Today, CO_2_ inhalation is commonly used as a diagnostic tool for PD (Battaglia and Perna, 1995; Gorman et al., 2001; Hoppe et al., 2012). An increased CO_2_ response is acknowledged as a distinctive biomarker of this population and remains a central readout of PD that is easily and non-inferentially modeled in the laboratory. Furthermore, PD patients show an abnormally elevated respiratory variability owing to excessive sighing and an increased rate of apnea both during sleep and wakefulness (Stein et al., 1995; Bystritsky et al., 2000; Abelson et al., 2001; Nardi et al., 2009; Garbarino et al., 2020; Feinstein et al., 2022). The sum of these physiological symptoms indicate that both the regulation and perception of breathing are dysfunctional in PD patients (Gorman et al., 2000; Sinha et al., 2000; Katzman et al., 2002; van Duinen et al., 2007; Abelson et al., 2008; Nardi et al., 2009; Abelson et al., 2010; Grassi et al., 2013).

In North America and Europe, PD affects ∼5% of the general population (Hoppe et al., 2012; Meng and D'Arcy, 2012; Bandelow and Michaelis, 2015) and its sexual dimorphism is striking: the prevalence rate of women who have PD or with excessive physiological and behavioral responses to CO_2_ inhalation is 2–3 times that of men (Wilhelm and Roth, 2001; Pigott, 2003; Donner and Lowry, 2013). The incidence of PD rises at puberty (Reardon et al., 2009) and in young adolescent girls, pubertal stage predicts panic attack occurrence (Hayward et al., 1992). Furthermore, the panicogenic effects of CO_2_ inhalation are highest during the pre-menstrual phase (Nillni et al., 2017), thus indicating that, by comparison with healthy subjects, women suffering from PD are more sensitive to the sudden drop in ovarian hormones taking place during the last days of the luteal phase (Reardon et al., 2009; Nillni et al., 2011; Nillni et al., 2017). Cyclic fluctuation in ovarian hormones is a normal physiological process, but in a subpopulation of women, they contribute to the onset of PD and its recurrent exacerbations (Reed and Wittchen, 1998; Gorman et al., 2001; Lovick, 2014). Thus, elucidating why hormonal fluctuations can lead to excessive responsiveness to CO_2_ offers a unique opportunity to gain insights into the neuroendocrine mechanisms regulating this key aspect of respiratory control and the pathophysiology of respiratory manifestations of PD.

## 3 Early life stress and PD-related respiratory disturbances in rodents and humans

In mammals (including humans), exposure to adversities during early life alters brain development and is a significant risk for disease (Graham et al., 1999; Buitelaar et al., 2003; Fumagalli et al., 2007; Shonkoff et al., 2009; Charil et al., 2010). Conditions such as maternal depression, unstable parental environment, and special medical care at birth that interfere with mother-infant interactions are stressful to the infant. The sum of current data from clinical and basic research indicates that these forms of early life stress predispose to behavioural and cognitive disorders as well as excessive CO_2_ sensitivity and PD that emerge at adolescence, especially in females (Battaglia et al., 1995; Gunnar, 2003; Battaglia et al., 2009; Shonkoff et al., 2009; D'Amato et al., 2011). Thus, the deleterious impacts of early life stress remain latent and are revealed by the rise in ovarian function that takes place at puberty; subsequent fluctuations of ovarian hormones exacerbate PD-related respiratory symptoms in a cyclic fashion. To gain insight into the basic neuroendocrine mechanisms of PD, repeated cross fostering (RCF) and neonatal maternal separation (NMS) have been use in mice and rats (respectively) as clinically relevant models of early life stress and using the ventilatory response to CO_2_ as a main physiological outcome (Battaglia et al., 2014).

**FIGURE 1:**
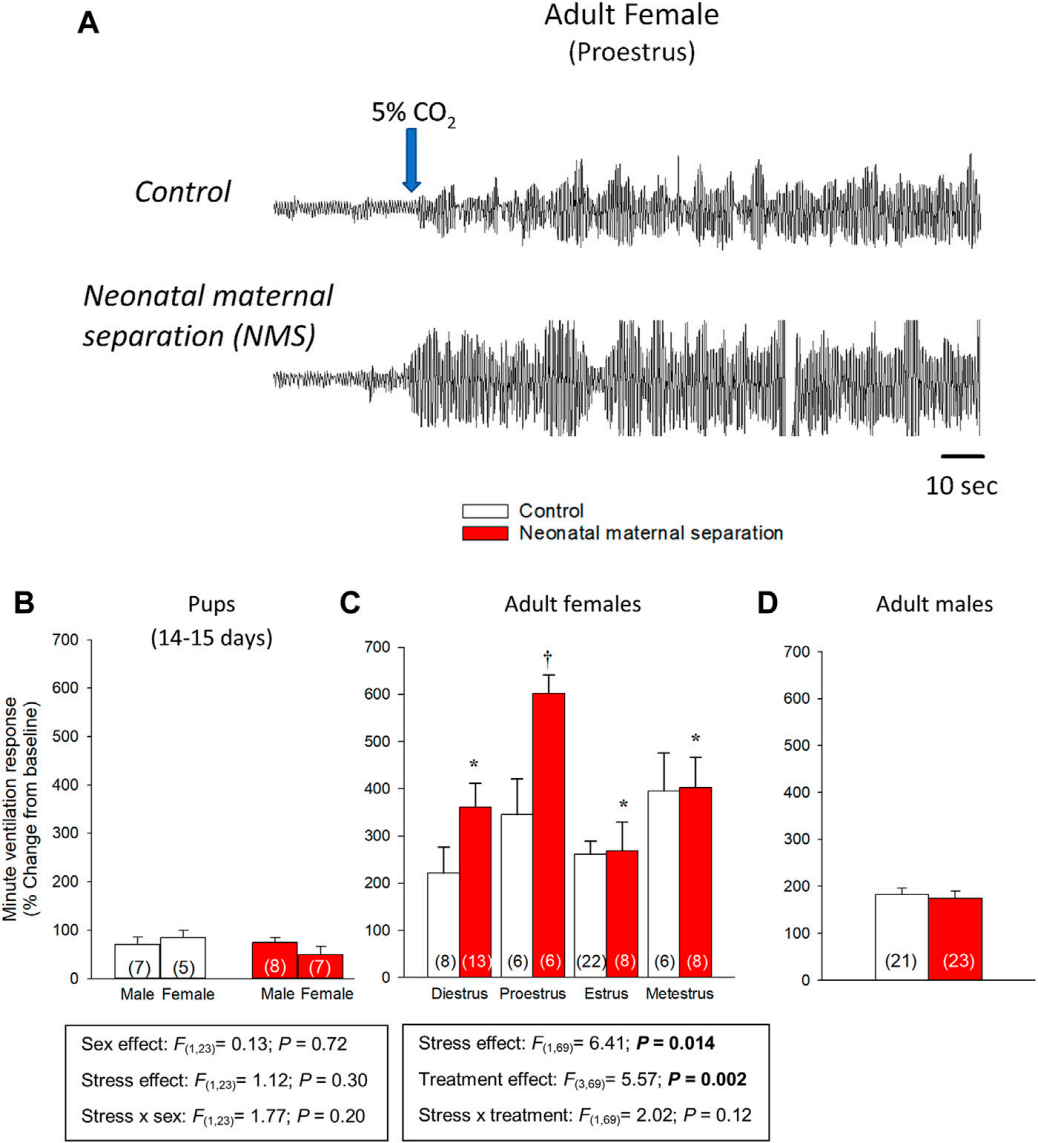
Influence of neonatal stress and reproductive status on the magnitude of ventilatory response to CO_2_ in rats. **(A)** Original plethysmography recordings comparing ventilatory activity at rest and upon exposure to hypercapnia (5% CO_2_ in air). Recordings were obtained from adult rats during the proestrus phase; females were either raised under standard conditions (top trace) or subjected to neonatal maternal separation (bottom trace; 3 h/day, postnatal days 3–12). Blue arrow indicates the onset of the exposition to 5% CO_2_ for 10 min. Comparison of the minute ventilation responses to hypercapnia between control rats (white bars) and rats subjected to neonatal maternal separation (NMS; red bars). Data expressed as percent change from baseline (room air) in **(B)** pups, adult **(C)** females and **(D)** males. Data from males are from (Genest et al., 2007; Tenorio-Lopes et al., 2017); they are reported for comparison and were not included in the statistical analyses. Data are reported as means ± SEM * indicates a value significantly different from corresponding proestrus value at *p* < 0.05; † indicates a value significantly different from corresponding control value at *p* < 0.05. Adapted with permission from (Tenorio-Lopes et al., 2020).

The hypercapnic ventilatory response (HcVR) changes significantly during development (Putnam et al., 2005; Tenorio-Lopes et al., 2020) and progressive increase in the expression TASK 1 and TASK 2 channels in the hypothalamus likely contribute to this process (Wang et al., 2021); however, the molecular signal initiating their expression remains unknown. In sexually mature mammals (including humans), sex-based differences in the intensity of the CO_2_ response are well documented but are highly heterogeneous (Gargaglioni et al., 2019). In mice, RCF augments the HcVR of pups (P16-20) and adults mainly by augmenting the tidal volume response; however, this effect is similar in both sexes (D'Amato et al., 2011; Luchetti et al., 2015; Cittaro et al., 2016; Battaglia et al., 2018). In adult C57BL6 mice, NMS elicits a modest increase of the HcVR only in females (Elliot-Portal et al., 2021). In rats, the ventilatory response to 5% CO_2_ of pre-pubertal rat pups (P14 - P15) is very weak with no evidence of sex- or NMS-related effects (Tenorio-Lopes et al., 2020). At adulthood, the HcVR of control (non-stressed) male and female rats is similar, but the effects of NMS on the CO_2_ response differ strikingly between sexes in ways that are very similar to clinical observations of PD. Specifically, the minute ventilation response to CO_2_ inhalation (5% CO_2_; 10 min) of NMS females is 60%–80% larger than controls and NMS-related increase of the CO_2_ response is ***i***) sex-specific (limited to females), ***ii***) peaks during proestrus, and ***iii***) is not observed prior to puberty (Genest et al., 2007; Kinkead et al., 2009) (Figure 1). These differences in the developmental and sex-specific effects of early life stress on the HcVR of mice and rats likely reflect inter-species differences in stress responses of rodents (Beery and Kaufer, 2015). Regardless, both models have advanced our comprehension of the pathophysiology of PD. What follows is a summary of the main mechanisms that contribute to stress-related increase in CO_2_ response.

## 4 Early life stress alters non-medullary structures with CO_2_ sensing properties

### 4.1 The amygdala

**FIGURE 2:**
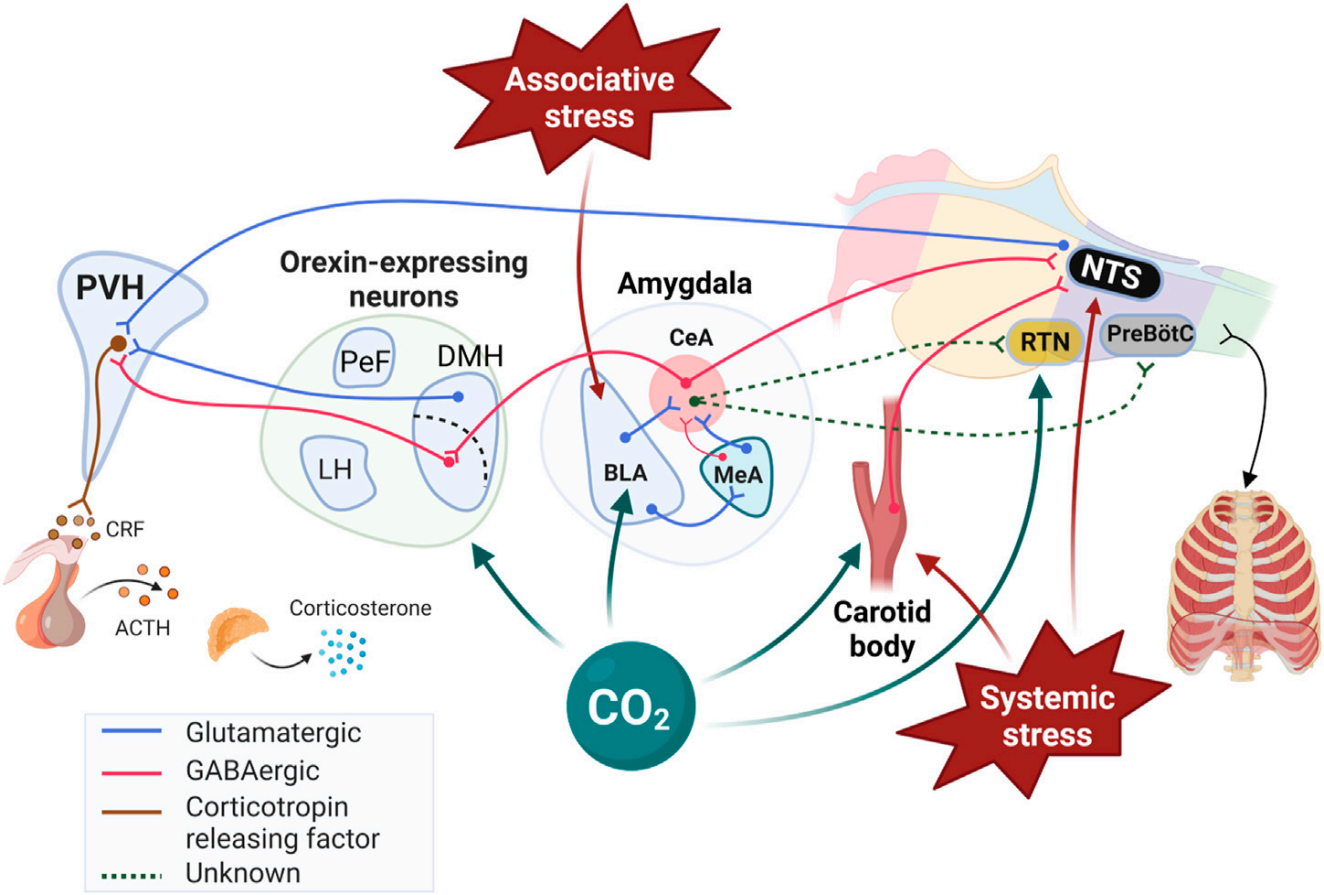
Schematic representation of stress-related neuronal inputs and CO_2_ chemosenstivity in cardiorespiratory control. While retrotrapezoid nucleus (RTN) of the medulla is well established as a highly sensitive to CO_2_/H^+^ responsible for fine respiratory adjustments, the Figure 1) highlights other “non-medullary” sites that respond to CO_2_/H^+^, 2) identifies the pathways by which they initiate significant cardiorespiratory and behavioral responses, and 3) illustrates how these structures are part of the neural network initiating and regulating the response to stress. Note that the interactions between the PVH and medullary structures regulating breathing are not shown for simplicity. The review by (Zhang et al., 2021) provided valuable informations on network and the neurotransmitters contributing to those interactions. PVH: paraventricular nucleus of the hypothalamus; BLA: Basolateral amygdala; CeA: Central nucleus of the amygdala; MeA: Medial nucleus of the amygdala; ORX; Orexin producing neurons; DMH: Dorsomedial hypothalamic nucleus; PeF: Perifornical area; LH: Lateral hypothalamus; NTS: Nucleus of the solitary tract; CRF: Corticotropin releasing factor; ACTH: Adrenocorticotropic hormone; PreBötC: Pre-Bötzinger complex. Created with BioRender.com.

Within the medial temporal lobes, the amygdalar complex is responsible for perception and processing of stimuli; it also initiates and terminates emotional reactions (Marek et al., 2013). Briefly, this complex is composed of three main structures: the medial amygdala (MeA), the central amygdala (CeA) and the basolateral amygdala (BLA) (Figure 2). The basolateral part of the amygdala (BLA) is of great interest to this mini-review because it has inherent CO_2_ sensing properties; much like “classical” CO_2_-sensing neurons of the medulla, BLA neurons can detect CO_2_ (Ziemann et al., 2009). While direct comparisons are not possible, we can estimate that the CO_2_/H^+^ sensitivity of BLA neurons is ∼ 10 times less than that of the retrotrapezoid nucleus (RTN), the main CO_2_ sensing structure in respiratory control (Guyenet et al., 2019). Nonetheless, CO_2_-induced stimulation of the BLA elicits emotional and physiological responses associated with fear and panic-related states (Ziemann et al., 2009). The BLA interacts with the medial amygdala (MeA) that regulates innate emotional behaviors; it relays olfactory information to hypothalamic nuclei involved in reproduction and defense behaviors. Interestingly, sex-based differences in anatomy, laterality, function, and sensitivity to steroid hormones of the MeA are well documented in humans and rodents (Rodrigues et al., 2009; Cahill, 2010; Goldstein et al., 2010; Edelmann and Auger, 2011; Buss et al., 2012). Both regions project to the central amygdala (CeA) (Keshavarzi et al., 2014), which is the amygdala’s output pathway because it initiates autonomic and respiratory responses (Veening et al., 1984). The CeA projects directly onto rhythmogenic neurons of the pre-Bötzinger complex, the nucleus of the solitary tract (NTS), and the RTN (Petrov et al., 1995; Rosin et al., 2006; Ulrich-Lai and Herman, 2009; Yang et al., 2020). Furthermore, stimulation of the CeA excites the inspiratory cycle (Harper et al., 1984). In our studies using *c-fos* mRNA as a marker of neuronal activation, the CeA has emerged as an important candidate in the initiation of an excessive ventilatory response to CO_2_ in NMS, especially in female rats (Kinkead et al., 2014).

Owing to its role in the regulation of emotional reactions, the amygdala contributes to the pathogenesis of anxiety (Feinstein et al., 2022); however, observations made on patients with Urbach–Wiethe disease, a rare genetic disorder leading to focal bilateral amygdala lesions, have raised questions concerning its contribution to PD. Briefly, Urbach–Wiethe patients show no fear and avoidance behavior to external threats such as snakes, tarantulas, and a range of traumatic life events, but exhibit a significantly higher rate of CO_2_-evoked fear and panic than a sample of demographically-matched healthy participants (Feinstein et al., 2013; Feinstein et al., 2022). In mice, electrolytic lesions of the amygdala inhibited fear-like behavior (freezing); however, the ventilatory response was not tested (Taugher et al., 2020). In pediatric subjects, electrical stimulation of the medial subregion of the basal nuclei, cortical and medial nuclei induces apnea (Rhone et al., 2020); similar procedures in the lateral and basolateral amygdala of adults also results in respiratory inhibition (Dlouhy et al., 2015). The fact that these patients do not notice respiratory arrest or report dyspnea indicate that their ability to perceive the rise in CO_2_ is blunted; the inverse relationship between CO_2_ activation of the MeA and the HcVR of male rats is in line with this observation (Tenorio-Lopes et al., 2017). Although compelling, this interpretation requires caution because amygdalar lesions in humans and mice were heterogeneous and clinical studies generally involve a limited number of participants. Nonetheless, the sum of these observations ***i***) highlights an important neural distinction between “external” threats conveyed via visual and auditory pathways, *versus* threats conveyed through “internal” sensory channels (e.g., chemoreceptive); ***ii***) suggests that rather than inducing panic, the amygdala inhibits it, especially when it is evoked by an internal threat such as elevated levels of CO_2_, and ***iii***) this inhibition likely originates from a subpopulation of CeA neurons considering that as discussed previously, the CeA is generally acknowledged for its stimulatory influence on breathing.

### 4.2 Orexin neurons

**FIGURE 3:**
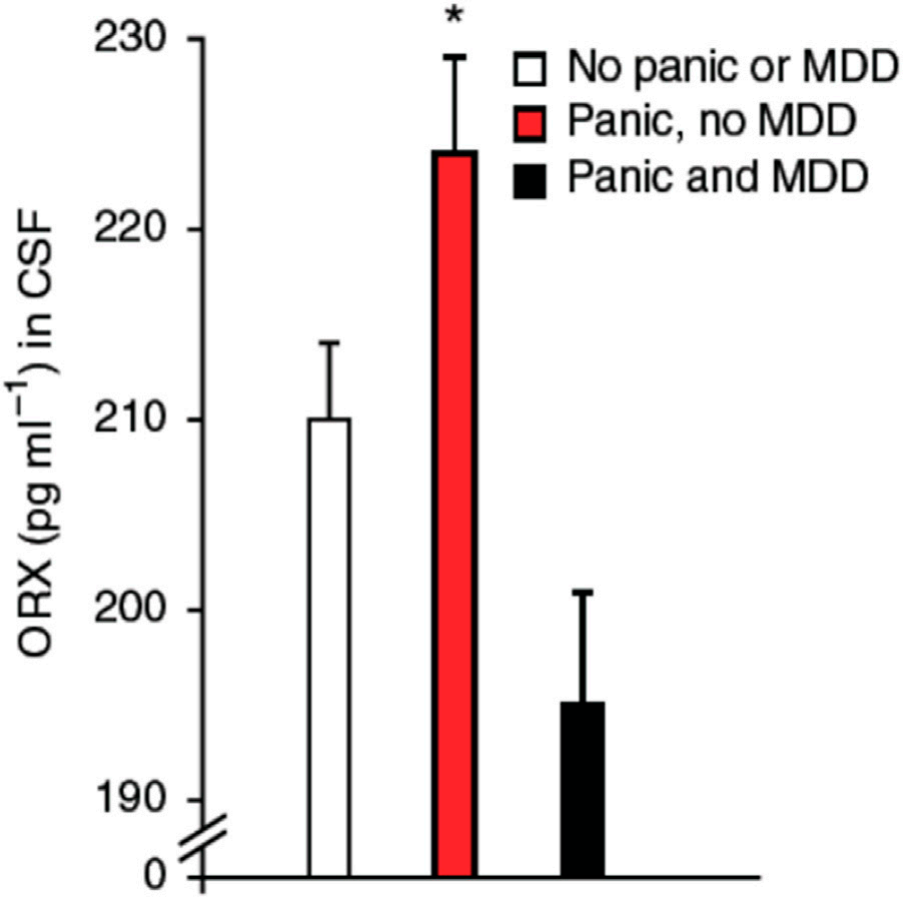
Orexin concentrations in cerebrospinal fluid (CSF) obtained by lumbar puncture in subjects with panic anxiety with or without major depressive disorder (MDD). Subjects who presented with acute suicidal behavior were systematically assessed for psychiatric symptoms utilizing the comprehensive psychopathological rating scale (CPRS), where item 3 (inner tension) assesses panic anxiety. A threshold cut off at 1.5 on this item was used to define a subject as having significant panic symptoms. All subjects with substance abuse and traces of anti-depressive, neuroleptic or anxiolytic medication in the blood were excluded from the analysis. Subjects with panic anxiety without MDD (n = 12); subjects with both panic and co-morbid MDD (n = 13); and subjects without panic, without MDD (n = 28). Data are reported as means ± SD; * indicates significant differences from other groups, using Kruskall Wallis ANOVA (*p* = 0.004); and two-tailed Mann-Whitney U-test (subjects with panic and MDD, *p* = 0.002; subjects without panic, *p* = 0.01). Reproduce with permission from Johnson et al. (2010).

Orexins A and B (ORX; also known as hypocretins) are regulatory peptides produced by neurons located in the dorso-medial, perifornical, and lateral hypothalamus (DMH, PeF, and LH, respectively). Orexin neurons have extensive projections throughout the central nervous system; however, the organisation of this system is dichotomous: LH neurons stimulate motivated behaviors such as appetite for food and other rewards such as abused drugs (Harris and Aston-Jones, 2006), whereas neurons of the PeF and DMH act in parallel to influence arousal, sleep/wake states, and cardiorespiratory function (Johnson et al., 2010; Nattie and Li, 2012; Ciriello et al., 2013; Barnett and Li, 2020). Interestingly, the DMH/PeF region was initially termed the “panic area” because its activation induced a “panic-like state” in experimental animals (DiMicco et al., 2002; Díaz-Casares et al., 2009). Today, a “hyperactive” ORX system is a leading hypothesis in the pathophysiology of PD (Johnson et al., 2010; Abreu et al., 2020). This is based on the fact that ORX levels in the cerebrospinal fluid of PD patients is elevated by comparison with healthy subjects (Johnson et al., 2010) (Figure 3) and in adult rats, previous exposure to early life stress (maternal deprivation/separation) augments ORX_A_ levels in hypothalamus extracts (Feng et al., 2007; Tenorio-Lopes et al., 2020). Furthermore, PD patients show abnormal levels of expression the HCRTR1 gene which encodes for ORX_1_ receptors (Johnson et al., 2010; Gottschalk et al., 2019).

**FIGURE 4:**
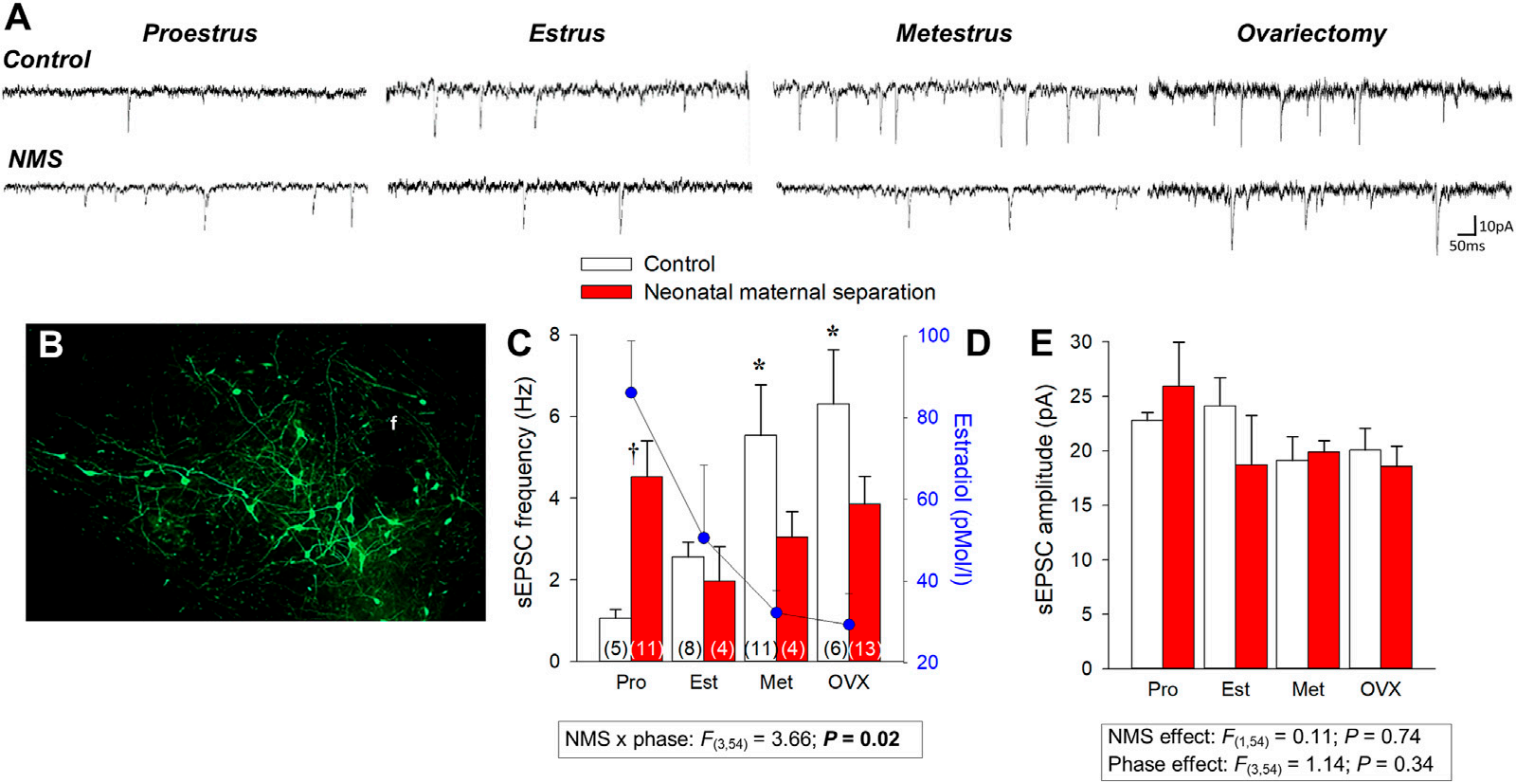
Neonatal maternal separation stress (NMS) reduces the spontaneous excitatory postsynaptic currents (sEPSC) recorded in GFP-labeled orexin neurons in response to changes in 17β-estradiol (E_2_) level in intact and ovarectomized rats. **(A)** Comparison of sEPSC recordings from orexin neurons between cells during different phase of the estrus cycle and 2 weeks following ovariectomy (OVX); tissue slices originated from females raised under control conditions (top traces) or subjected to NMS (bottom traces; 3 h/day, postnatal days 3–12). **(B)** Photomicrograph illustrating GFP-labeled orexin neurons; the fornix (f) is shown as a landmark. **(C)** Population data of EPSC frequencies recorded during 3 distinct phases of the estrus cycle and following OVX. **(D)** Baseline E_2_ values are reported for comparison; values from NMS and controls were pooled since they are not statistically different. **(E)** Reports sEPSC amplitudes. Data are reported as means ± SEM; * *p* < 0.05 compare to corresponding proestrus value; † *p* < 0.05 compare to corresponding control. *Adapted from* Tenorio-Lopes *et al* (2020).

**FIGURE 5:**
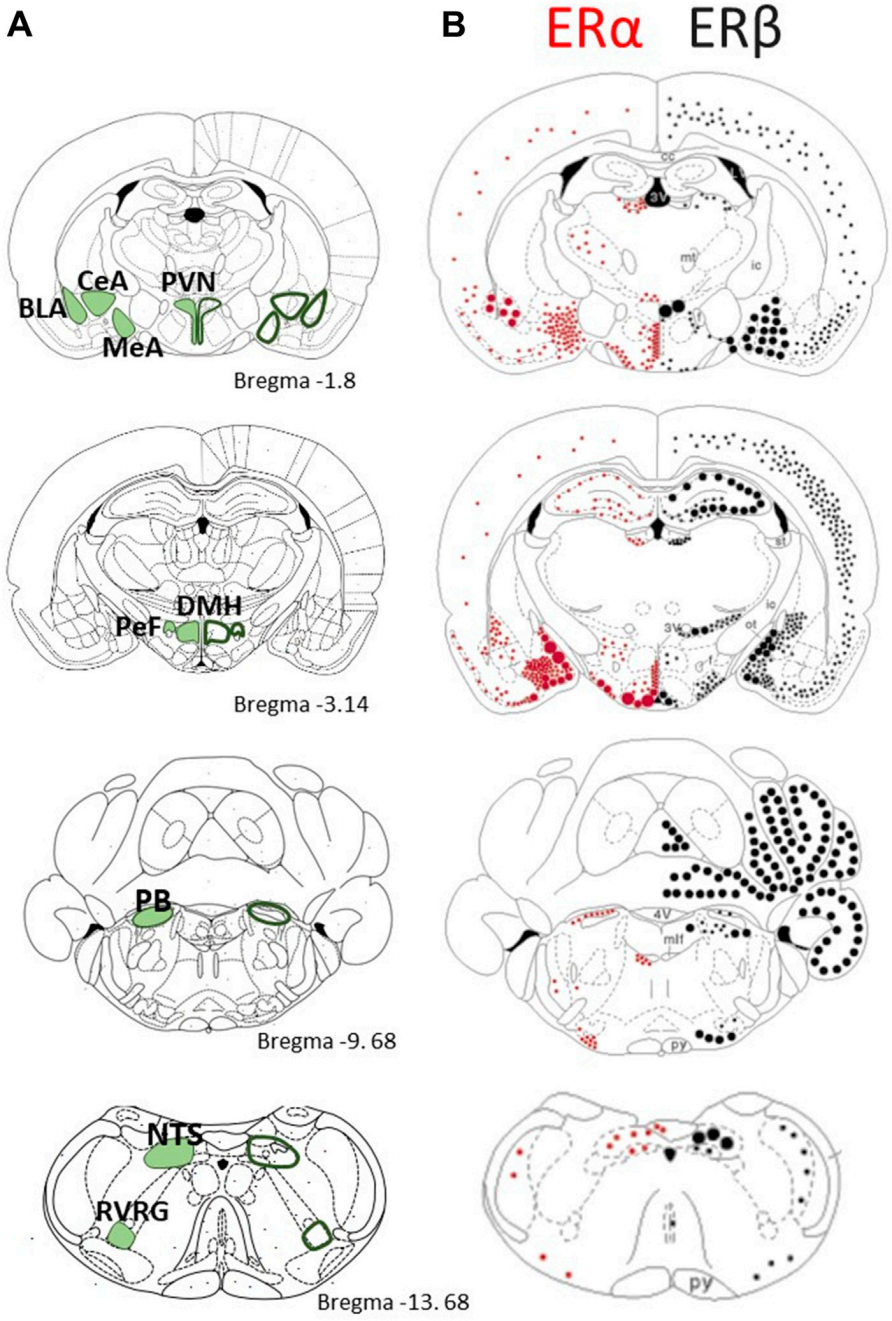
Comparison of the distribution of estrogen receptors α and β in brain regions that are responsive to CO_2_/H^+^ sensing and/or contribute to the stress response **(A)** Schmatics on the left present a series of coronal section modified from the rat brain atlas of Paxinos and Watson (1998) with emphasis on key structure with CO_2_ sensing properties or with established roles in respiratory control; the stereotaxic reference (distance from bregma) is indicated. **(B)** Schematics on the right present the distribution of ERα (red dots) and ERβ (black dots) mRNA in the rat brain. Small dots represent 1–5 labeled cells; medium dots 6–10 labeled cells; large dots represent approximately 50 labeled cells. Adapted with permission from Shughrue et al., 1997. PVH: Paraventricular nucleus of the hypothalamus; BLA: Basolateral amygdala; CeA: Central nucleus of the amygdala; MeA: Medial nucleus of the amygdala; DMH: Dorsomedial hypothalamic nucleus; PeF: Perifornical area; PB: Parabrachial nucleus; NTS: Nucleus of the solitary tract; RVRG: Rostral ventral respiratory group.

Orexin acts on two receptors (ORX_1_ and ORX_2_) and their expression in the pontomedullary areas of the autonomic and respiratory network overlap partially (Marcus et al., 2001). ORX_A_ can bind to both receptors whereas ORX_B_ binds primarily to ORX_2_ (Carrive and Kuwaki, 2017). The fact that the basal respiratory activity of ORX-knock out mice is similar to that of wild-type indicates that ORX neurons have limited impacts on breathing at rest (Nakamura et al., 2007; Berteotti et al., 2020) and while there is evidence indicating that deletion of ORX neurons increases apneic events during sleep (Nakamura et al., 2007), this effect is not always observed (Berteotti et al., 2020). However, activation of ORX neurons potentiates chemoreflexes and there is growing evidence indicating that ORX neurons have CO_2_-sensing properties (Gestreau et al., 2008; Li and Nattie, 2014; Carrive and Kuwaki, 2017). In mice, exposure to CO_2_ (10% CO_2_; 3 h) augments the number of *c-FOS* immunolabeling in ORX_A_ expressing neurons of the PeF and DMH (but not LH) (Sunanaga et al., 2009). Results from electrophysiological experiments provide more direct support as they show that acidification of the extracellular milieu increases intrinsic excitability and firing rate of ORX cells, whereas alkalinization depresses it. Furthermore, this effect involves acid-induced closure of K^+^ channels in the orexin cell membrane (Williams et al., 2007). These responses resemble those of known chemosensory neurons; however, the authors did not specify the specific location of the populations of ORX neurons that were recorded. Regulation of ORX neurons is greatly influenced by gonadal hormones and data show that the intensity of expression of ORX_1_ receptors in the hypothalamus parallels ovarian hormone levels. In rats, their expression peaks during the proestrus phase (Silveyra et al., 2009). As a result, Grafe and Bhatnagar proposed that the ORX system is fundamental to sex-based differences in stress-related neurological disorders such as PD (Grafe and Bhatnagar, 2018). Estradiol (E_2_) is of great interest in this context because E_2_ levels rise during proestrus and its inhibitory actions on ORX neurons reduce their responsiveness to stress (Shors et al., 2001). To evaluate the role of E_2_ in regulating ORX neurons we first used immunohistochemistry and data convincingly show that under resting conditions, OVX augments the ratio of *c-FOS*/ORX_A_ immunolabeled cells in control females, especially in the DMH. Conversely, OVX had no significant effect in NMS females because the number of labeled cells was already elevated in intact females (Tenorio-Lopes and Kinkead, 2021). We then used whole cell recording to evaluate how changes in E_2_ across the estrus cycle affects synaptic inputs converging onto ORX neurons and our results showed that NMS reduces E_2_-mediated inhibition of ORX neurons (Figure 4). During proestrus, the excitatory post-synaptic current (EPSC) frequency of control females was the lowest whereas in NMS females the frequencies were the highest observed in this group. These observations provide a plausible explanation for the higher *c-FOS*/ORX_A_ immunolabeling, greater ORX_A_ levels measured in hypothalamic extract, and the high efficacy of systemic administration of the selective ORX_1_ receptor antagonist SB-334867 (15 mg/kg; IP) at reducing the HcVR of NMS female rats. Of note, this drug-induced attenuation of the HcVR was most important during the proestrus phase; in controls, this treatment had no significant effect on the HcVR, regardless of the estrus phase (Tenorio-Lopes and Kinkead, 2021). The sum of these data indicates that stress-induced disruption of E_2_ signalling is an important mechanism in a rat model of PD and that ORX neurons is an important site of action (Figures 2, 5).

## 5 Early life stress and its impacts on other mechanisms contributing to CO_2_ sensing

### 5.1 Acid-sensing ion channels (ASICs)

The ventilatory response to CO_2_ is determined by multiple chemosensory structures with specialised capacity for detecting changes in CO_2_/H^+^ in their vicinity that project to respiratory neurons to initiate a robust increase in breathing. Acid-sensing ion channels (ASICs) are widely expressed in the brain, including the ventrolateral medulla, where they play a pivotal role in driving CO_2_/H^+^ chemosensing and triggering emotional and physiological responses (Song et al., 2016). Regardless of their biological sex, PD patients show variation of the ACCN2 gene, the human ortholog of the *Asic1a* (Smoller et al., 2014). Consistent with human data, mRNA transcript analysis of the brainstems of male and female mice show heightened ASIC expression in RCF exposed animals (Cittaro et al., 2016); although ASICs are comprised of multiple subunits the authors presumably refer to ASIC1A but this was not specified.

The fact that inactivation of ASIC channels with amiloride attenuates the HcVR of RCF animals but not controls strongly suggests that overexpression of these ion channels is an important mechanism in the abnormal respiratory phenotype associated with PD (Battaglia et al., 2018). However, the fact that amiloride has non-specific effects on a number of other receptors and transporters needs to be considered.

### 5.2 The carotid bodies

Strategically located at the bifurcation of the carotid arteries, the carotid bodies are main sensors of O_2_ levels in the arterial blood; however, they also respond to changes in arterial CO_2_/H^+^ (Iturriaga et al., 2021). They project to the medulla where they provide powerful chemosensory signals to the respiratory network. Because carotid body stimulation by potassium cyanide injection stimulates fear and escape responses (Schimitel et al., 2012), we determined whether these chemosensors contribute to the excessive HcVR of NMS females. To do so, we compared the responsiveness of the carotid bodies to changes in O_2_ and CO_2_ using an *ex vivo* preparation and the results convincingly showed that NMS does not affect peripheral CO_2_ sensing in either sex (Soliz et al., 2016). We therefore concluded that anomalies in the CO_2_ chemoreflex takes place within the brain.

We first evaluated the contribution of the central nervous system (CNS) to this phenotype using anesthetised rats (Dumont and Kinkead, 2010; Dumont et al., 2011; Dumont and Kinkead, 2011), but this approach eliminated the NMS-induced increase of the ventilatory response to CO_2_ reported in awake females (Dumont et al., 2011). This led us to propose that NMS disrupts anesthesia-sensitive structures responsible for the cognitive and/or emotional perception of the CO_2_ stimulus (Dumont et al., 2011). This inference was first based on the notion that CO_2_ is both a systemic and associative stressor. In other words, CO_2_ is capable of stimulating both physiological (*i.e.,* respiratory) reflexes via conventional pathways and strong emotional and associative reactions, such as fear and escape responses that, in turn, further stimulate breathing (Schenberg, 2016). These observations and the current background knowledge brought our attention to the amygdala.

### 5.3 Microglia

Microglia are the immune cells of the brain that are mainly known for scavenging the CNS for infectious agents, damaged or unnecessary neurons and synapses. However, there is growing evidence indicating that uncoupling neuron–microglia interactions alters neuroplasticity and contributes to anxiety- or depressive-like behaviors (Koo and Wohleb, 2021). Microglia express cell death–associated gene-8 (TDAG8), an acid-sensing G-protein coupled receptor which is necessary for full expression of CO_2_-evoked fear (Vollmer et al., 2016). Specifically, freezing and blood pressure responses to CO_2_ inhalation (5% CO_2_; 10 min) of TDAG8 deficient mice are lower than those reported in wildtype animals; however, the HcVR does not differ between genotypes (Vollmer et al., 2016). Subsequent experiments demonstrated that upon CO_2_ exposure, microglia release the proinflammatory cytokine IL-1β which then activates neurons. Quantification of microglial activation and electrophysiological assessment of the CO_2_ responses were performed in the subfornical organ, a circumventricular organ that lacks a blood brain barrier. Based on comparisons of the cell’s firing rate response of subfornical neurons, the sensitivity to CO_2_/H^+^ is ∼10 times less than that reported for the RTN (Guyenet et al., 2019). It was argued that blood-born compounds can have access to the CNS via this route such that this structure acts as an integrative site for the maintenance of homeostasis (Vollmer et al., 2016). This explanation raises the possibility that the area postrema plays a similar role in respiratory manifestations of PD. The area postrema is a medullary circumventricular organ with chemosensing properties located above the NTS; it expresses inward rectifier K^+^ channels (Kir) associated with CO_2_ chemosensitivity (Wu et al., 2004) and projects to the RTN, a key medullary structure in CO_2_ chemodetection (Rosin et al., 2006). This idea is certainly worth exploring.

### 5.4 Estrogens

Gonadal hormones are “the usual suspects” in mechanistic studies aiming to explain sex-based differences in physiological function. The contribution of 17β-estradiol (E_2_) is intriguing owing to its multiple and heterogeneous influences on the stress response. On the one hand, the onset of PD-related respiratory disturbances coincides with the rise in circulating E_2_ at puberty and reports of panic attacks in *some* women receiving E_2_-replacement therapy (Price and Heil, 1988; Hayward et al., 1992). This is consistent with the view that E_2_ is a potent stimulant of the hypothalamic pathways regulating the stress response; female rats in proestrus (high estradiol, high progesterone) and estrus (recent exposure to peak estradiol), have elevated basal and stress induced corticotropic hormone (ACTH) and corticosterone (Viau and Meaney, 1991; Heck and Handa, 2019). On the other hand, E_2_-replacement therapy may reduce panic symptoms in women and transdermal E_2_ treatment in menopausal women has been reported to blunt the acute stress response (Lindheim et al., 1992; Chung et al., 1995). In rats, E_2_-supplementation of ovariectomized females can reduce the response to chronic recurrent stress by attenuating the output of the paraventricular nucleus of the hypothalamus (PVN) (Gerrits et al., 2005). The sum of these observations underlies the view that E_2_ has anxiolytic properties (Österlund, 2010; Borrow and Handa, 2017). Thus, there is no clear consensus and these apparent discrepancies reflect challenges commonly encountered in stress studies in which the responses vary depending of the intensity, nature, and duration of the challenge used. Furthermore, the sex, species, age/ovarian status of the female along with environmental factors such as nutrition contribute to the variability of estrogen’s actions (Borrow and Handa, 2017; Heck and Handa, 2019). As we discuss below, the two main E_2_ receptors (ERα and ERβ) have opposing actions on network function, such that slight changes in their relative expression can alter E_2_’s net effects and thus explain the heterogeneity in its effects (Kunte et al., 2014; Borrow and Handa, 2017).

E_2_ was initially shown to act via “classical” ERα and ERβ that are ligand-activated transcription factors influencing gene expression; however, both receptors are also expressed outside the nucleus where they induce non-genomic actions. E_2_ binds equally well to ERα and ERβ, but the two receptors are not functionally interchangeable; the differences in their localisation throughout the rodent brain support this functional divergence (Figure 5 **;** adapted from (Shughrue et al., 1997). Interestingly, the distribution of ERα and ERβ is similar between sexes (Hara et al., 2015), but the levels of expression are generally greater in females (Garcia-Segura et al., 2001). E_2_ also exerts rapid effects via membrane-bound G-protein estrogen receptors (GPERs); their discovery being more recent (1990s), the responses induced by GPERs are less documented (Hara et al., 2015; Barton et al., 2018). Together, these receptors allow E_2_ to alter the structure and function of neuronal networks via multiple mechanisms with time courses ranging from seconds to days (Evrard and Balthazart, 2004). We know for instance that E_2_ facilitates the transmission of electrical signals by promoting synaptic transmission via ERα. The concurrent actions of E_2_ on ERβ promote the formation of dendritic spines such that in the hippocampus, the spine density fluctuates with the estrus cycle and peaks on the day of proestrus (Woolley and McEwen, 1992; Tan et al., 2012), which is the phase when the largest CO_2_ response in NMS females were observed (Figure 1) (Tenorio-Lopes et al., 2020). E_2_ is strongly linked with anxiety disorders and a common view is that activation of ERβ is responsible for its anxiolytic effects whereas ERα initiate fear and anxiety-like behaviors (Walf and Frye, 2006; Frye et al., 2008; Borrow and Handa, 2017). Such generalisation requires caution, however, because each receptor type has distinct effects on glutamatergic and GABAergic signalling. The relative expression of each receptor type thus determines the balance between excitation and inhibition and E_2_’s net effect on a system (Woolley, 2007; Liu et al., 2008; Tan et al., 2012; Tian et al., 2013). Still, this balance is plastic and factors such as E_2_ levels and stress influence the relative expression of ERs. For instance, acute immobilization stress augments ERα immunolabeling in the PVN and medullary noradrenergic neurons (A2 area) of females (Estacio et al., 1996). Conversely, E_2_ generally reduces ERs in the hypothalamus (Simerly and Young, 1991; Garcia-Segura et al., 2001). GPERs also contribute to anxious phenotypes but their role remains unclear because opposing behavioral responses have been reported (Tian et al., 2013; Borrow and Handa, 2017).

Disruption of E_2_-signalling has therefore emerged as a key mechanism in anxiety disorders (Östlund et al., 2003; Albert et al., 2015) and although respiratory symptoms are an important feature of PD, our comprehension of the actions of E_2_ on the respiratory control system (including CO_2_ sensing) is still in its infancy, especially in females. Because female rats previously subjected to NMS closely replicate ontogenic and cyclic features of respiratory manifestations of PD, we took advantage of this model to further our understanding of the contribution of E_2_ on the ventilatory response to CO_2_.

**FIGURE 6:**
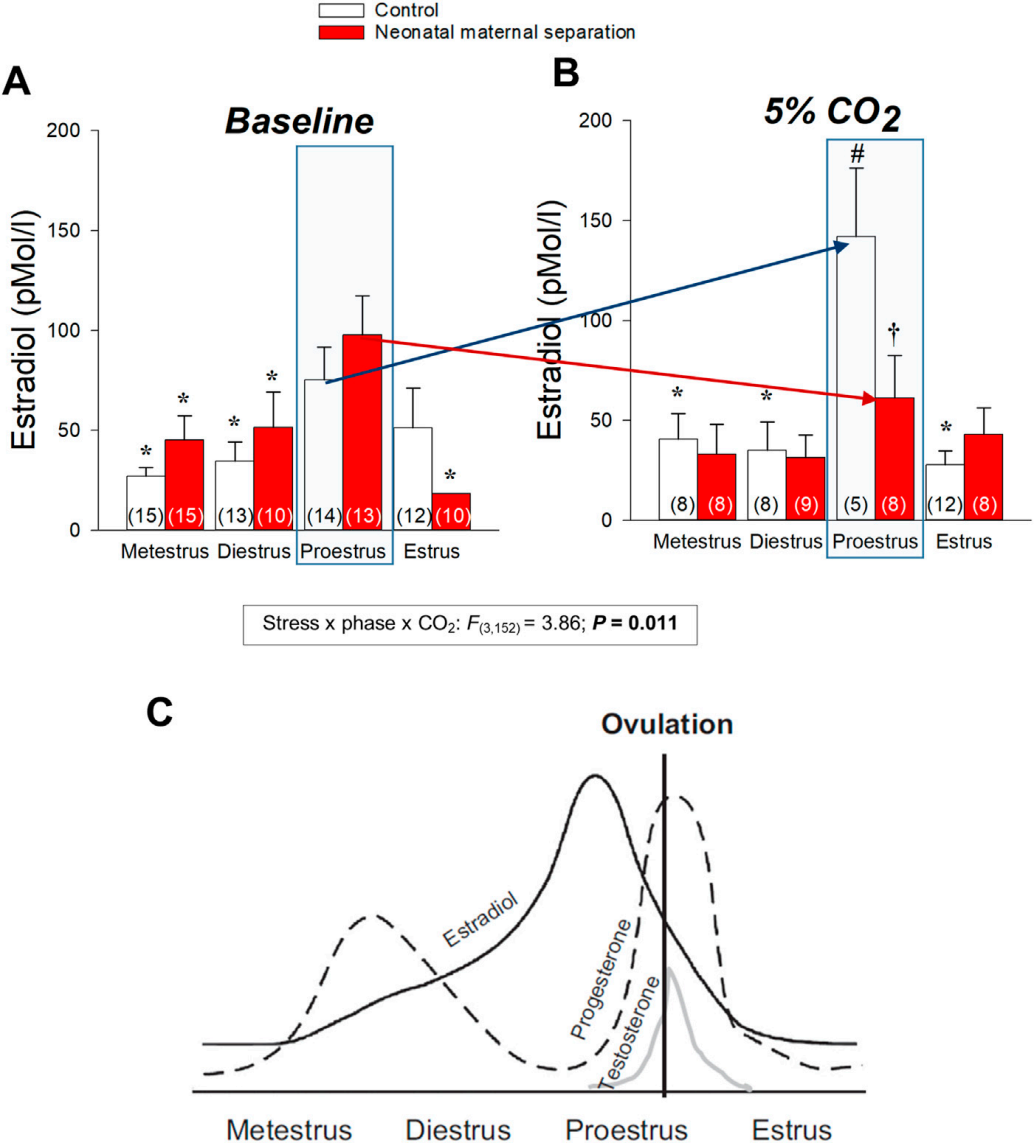
Neonatal stress disrupts the plasma 17β-estradiol (E_2_) response to CO_2_ inhalation of female rats. Plasma E_2_ levels measured across the different phases of the estrus cycle **(A)** in room air and **(B)** 30 min following exposition to 5% CO_2_ for 10 min. The numbers in brackets indicate the number of replicates in each group. **(C)** Schematic representation of the changes in estradiol, progesterone, and testosterone across the estrus cycle in rat. Repreoduc with persmission from (Pfaus et al., 2015). Data are reported as means ± SEM; * indicates a value different from corresponding proestrus value at *p* < 0.05; † indicates a value different from corresponsing control value at *p* < 0.05; # indicates a value signifincatly different from corresponsing baseline value at *p* < 0.05 compare to baseline value. Reproduced with permission from (Tenorio-Lopes et al., 2020).

**FIGURE 7:**
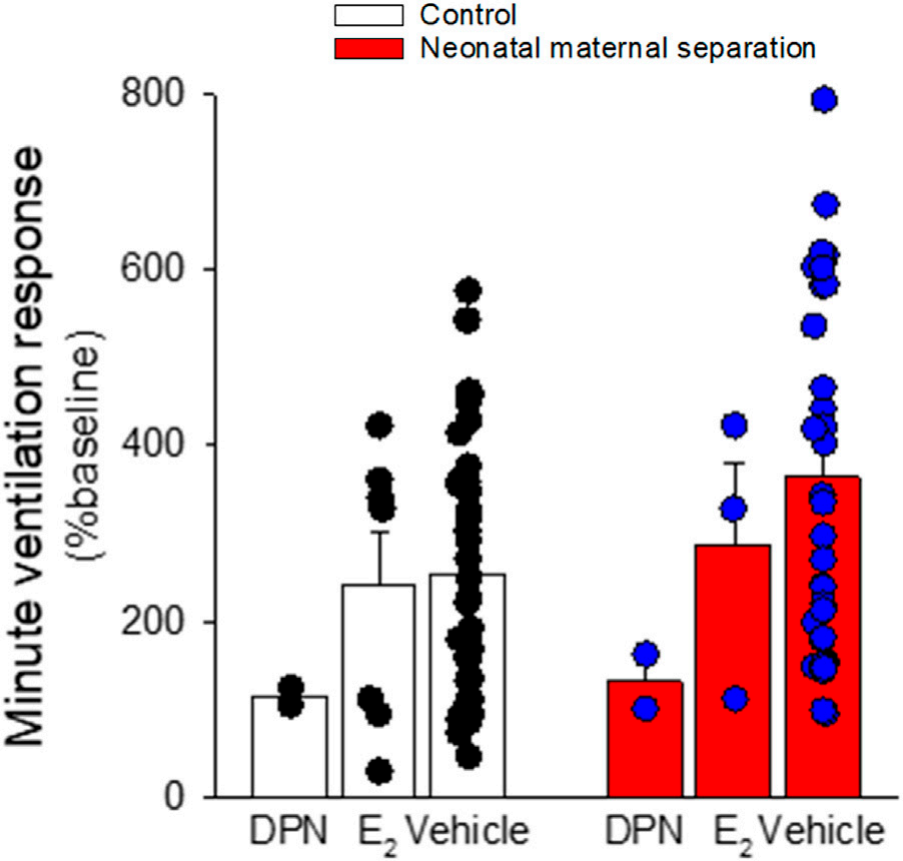
17-β estradiol supplementation attenuates the ventilatory response to the CO_2_ inhalation (5% CO_2_; 10 min) in female rats. Rats with intact ovaries either received vehicle, 17-β estradiol (40 μg/kg), or the ER-β agonist diarylpropionitrile (DPN; 0.1 mg/kg) 1 h before the onset of the experiment. Data are reported as means ± SEM; all phases of the cycle are combined. The low number of replicates in DPN treated rats did not allow proper statistical analysis.

Comparison of basal E_2_ and progesterone levels between NMS and controls across the estrus cycle does not indicate that NMS affects the gonadotropic axis at rest (Figure 6) (Dumont et al., 2011; Tenorio-Lopes et al., 2020). However, analysis of samples harvested following CO_2_ inhalation shows that this acute challenge stimulates E_2_ release during proestrus in controls but not in NMS females (Tenorio-Lopes et al., 2020) (Figure 6). We then noted that during proestrus, the intensity of the hyperventilatory response observed in NMS females was inversely proportional to E_2_ levels observed following CO_2_ exposure (Tenorio-Lopes et al., 2020). These data indicate that high E_2_ is a powerful inhibitor of the ventilatory response to CO_2_ but the E_2_ level achieved in NMS females is insufficient to prevent an excessive HcVR, especially during proestrus (Tenorio-Lopes et al., 2020). We then tested those conclusions by injecting E_2_ (3, 10, or 25 µg) in ovariectomized (OVX) females once per day every 4 days to restore E_2_ level within physiological range and mimic cyclic fluctuations. The last injection was performed ∼2 h prior to ventilatory measurements. Consistent with previous observations in rats, OVX reduced the HcVR (Marques et al., 2015), but the drop was greater in NMS females such that their HcVR (post-OVX) was comparable to that of controls with intact gonads (Tenorio-Lopes et al., 2020). Results from supplementation experiments clearly show that in NMS females, E_2_’s actions are biphasic with an increasing stimulatory effect until plasma levels reached the range observed during proestrus (∼150 pMol/l); higher doses no longer stimulated the HcVR. In contrast, E_2_’s influence on the HcVR of controls was limited. In a preliminary and unpublished experiment, we determined whether ERβ contributes to this process by testing the effect of acute IP injection of E_2_ (40 μg/kg) and the selective ERβ agonist diarylpropionitrile (DPN, 0.1 mg/kg) 1 h prior to CO_2_ inhalation test. Preliminary observations suggest that, at these doses, this treatment is more effective than E_2_ at reducing the HcVR, especially in NMS females (Figure 7). These results require further validation and raise questions about the stimulatory actions of a selective ERα agonist on the HcVR. Notwithstanding, E_2_ can be an important modulator of the neural pathways regulating the CO_2_ response; it would be interesting to determine whether those actions take place within “classical” medullary circuits or involve more rostral structures. As we discuss below, recent data revealed orexin producing neurons of the hypothalamus as key players in the process.

## 6 Conclusion and future directions

CO_2_ monitoring is essential to respiratory homeostasis and health; consequently, deciphering the cellular and molecular underpinnings of CO_2_ sensing and the neural networks driving reflexive responses has been a long-standing quest for physiologists. The presence of CO_2_ sensing neurons on the ventral surface of the medulla has been suspected since the 1960s and today, the RTN is firmly established as a primary structure in feedback regulation of breathing (Guyenet and Bayliss, 2022). The use of modern, “state of the art” approaches has led to important discoveries regarding the role and function of the RTN and we now know that this structure responds to very small changes in CO_2_/H^+^ to induce precise respiratory adjustments without producing any conscious aversive sensation (dyspnea), stress, or arousal (Guyenet and Bayliss, 2022). This mini-review and other contributions to this special issue demonstrate that other (non-medullary) brain regions are important contributors to central CO_2_ chemosenstivity (Nattie and Li, 2011). Figure 2 illustrates how these various structures interact to influence breathing and how E_2_ related signalling influences network function. While the evidence indicating that these structures can reflexively induce arousal and behavioral responses is compelling, further experiments are necessary to determine their specific contribution to respiratory control since the threshold for their activation seems greater than the RTN. Moreover, it is imperative to determine whether their response to CO_2_ is the result of a direct action of CO_2_/H^+^ or “network driven” changes. Although the contribution of these structures to homeostasis maybe limited under “standard” (healthy) conditions, their contribution to various respiratory disorders is convincing. For instance, CO_2_-induced arousal contributes to sleep fragmentation during sleep apnea (Kaur et al., 2017; Kaur and Saper, 2019; Kaur et al., 2020) and impairment of this arousal response may be important in the pathophysiology of sudden unexpected death in epilepsy, sudden infant death syndrome, and sleep apnea (Buchanan and Richerson, 2010; Smith et al., 2018; Buchanan, 2019). Here, our discussion focused on PD and the mechanistic studies performed in this context brings further support to this notion as they show that the excessive HcVR observed in stressed female rats reflect abnormal CO_2_ sensing taking place in structures near the hypothalamus.

## References

[B1] Abelson J. L. Khan S. Giardino N. (2010). HPA axis, respiration and the airways in stress-A review in search of intersections. Biol. Psychol. 84 (1), 57–65. 10.1016/j.biopsycho.2010.01.021 20144683

[B2] Abelson J. L. Khan S. Lyubkin M. Giardino N. (2008). Respiratory irregularity and stress hormones in panic disorder: Exploring potential linkages. Depress. Anxiety 25 (10), 885–887. 10.1002/da.20317 17557312

[B3] Abelson J. L. Weg J. G. Nesse R. M. Curtis G. C. (2001). Persistent respiratory irregularity in patients with panic disorder. Biol. Psychiatry 49 (7), 588–595. 10.1016/s0006-3223(00)01078-7 11297716

[B4] Abreu A. R. Molosh A. I. Johnson P. L. Shekhar A. (2020). Role of medial hypothalamic orexin system in panic, phobia and hypertension. Brain Res. 1731, 145942. 10.1016/j.brainres.2018.09.010 30205108

[B5] Albert K. Pruessner J. Newhouse P. (2015). Estradiol levels modulate brain activity and negative responses to psychosocial stress across the menstrual cycle. Psychoneuroendocrinology 59, 14–24. 10.1016/j.psyneuen.2015.04.022 26123902 PMC4492530

[B6] American-Psychiatric-Association (1994). Diagnostic and statistical manual of mental disorders: DSM-IV. Washington: American-Psychiatric-Association.

[B7] Ashhad S. Kam K. Del Negro C. A. Feldman J. L. (2022). Breathing rhythm and pattern and their influence on emotion. Annu. Rev. Neurosci. 45 (1), 223–247. 10.1146/annurev-neuro-090121-014424 35259917 PMC9840384

[B8] Bandelow B. Michaelis S. (2015). Epidemiology of anxiety disorders in the 21st century. Dialogues Clin. Neurosci. 17 (3), 327–335. 10.31887/DCNS.2015.17.3/bbandelow 26487813 PMC4610617

[B9] Barnett S. Li A. (2020). Orexin in respiratory and autonomic regulation, health and diseases. Compr. Physiol. 10, 345–363. 10.1002/cphy.c190013 32163209

[B10] Barton M. Filardo E. J. Lolait S. J. Thomas P. Maggiolini M. Prossnitz E. R. (2018). Twenty years of the G protein-coupled estrogen receptor GPER: Historical and personal perspectives. J. Steroid Biochem. Mol. Biol. 176, 4–15. 10.1016/j.jsbmb.2017.03.021 28347854 PMC5716468

[B11] Battaglia M. Bertella S. Politi E. Bernardeschi L. Perna G. Gabriele A. (1995). Age at onset of panic disorder: Influence of familial liability to the disease and of childhood separation anxiety disorder. Am. J. Psychiatry 152 (9), 1362–1364. 10.1176/ajp.152.9.1362 7653694

[B12] Battaglia M. Ogliari A. D’Amato F. Kinkead R. (2014). Early-life risk factors for panic and separation anxiety disorder: Insights and outstanding questions arising from human and animal studies of CO_2_ sensitivity. Neurosci. Biobehav. Rev. 46 (3), 455–464. 10.1016/j.neubiorev.2014.04.005 24793177

[B13] Battaglia M. Perna G. (1995). The 35% CO2 challenge in panic disorder: Optimization by receiver operating characteristic (ROC) analysis. J. Psychiatr. Res. 29 (2), 111–119. 10.1016/0022-3956(94)00045-s 7666379

[B14] Battaglia M. Pesenti-Gritti P. Medland S. E. Ogliari A. Tambs K. Spatola C. M. (2009). A genetically informed study of the association between childhood separation anxiety, sensitivity to co2, panic disorder, and the effect of childhood parental loss. Archives General Psychiatry 66 (1), 64–71. 10.1001/archgenpsychiatry.2008.513 19124689

[B15] Battaglia M. Rossignol O. Bachand K. D’Amato F. R. Koninck Y. D. (2018). Amiloride modulation of carbon dioxide hypersensitivity and thermal nociceptive hypersensitivity induced by interference with early maternal environment. J. Psychopharmacol. 0 (0), 101–108. 10.1177/0269881118784872 29968500

[B16] Beery A. K. Kaufer D. (2015). Stress, social behavior, and resilience: Insights from rodents. Neurobiol. Stress 1, 116–127. 10.1016/j.ynstr.2014.10.004 25562050 PMC4281833

[B17] Berteotti C. Lo Martire V. Alvente S. Bastianini S. Matteoli G. Silvani A. (2020). Effect of ambient temperature on sleep breathing phenotype in mice: The role of orexins. J. Exp. Biol. 223 (13), jeb219485. 10.1242/jeb.219485 32457059

[B18] Borrow A. P. Handa R. J. (2017). Estrogen receptors modulation of anxiety-like behavior. Vitam. Horm. 103, 27–52. 10.1016/bs.vh.2016.08.004 28061972 PMC5815294

[B19] Buchanan G. F. (2019). Impaired CO_2_-induced arousal in SIDS and SUDEP. Trends Neurosci. 42 (4), 242–250. 10.1016/j.tins.2019.02.002 30905388 PMC6512334

[B20] Buchanan G. F. Richerson G. B. (2010). Central serotonin neurons are required for arousal to CO_2_ . Proc. Natl. Acad. Sci. 107 (37), 16354–16359. 10.1073/pnas.1004587107 20805497 PMC2941296

[B21] Buitelaar J. K. Huizink A. C. Mulder E. J. de Medina P. G. R. Visser G. H. A. (2003). Prenatal stress and cognitive development and temperament in infants. Neurobiol. Aging 24, S53–S60. 10.1016/s0197-4580(03)00050-2 12829109

[B22] Buss C. Davis E. P. Shahbaba B. Pruessner J. C. Head K. Sandman C. A. (2012). Maternal cortisol over the course of pregnancy and subsequent child amygdala and hippocampus volumes and affective problems. Proc. Natl. Acad. Sci. 109 (20), E1312–E1319. 10.1073/pnas.1201295109 22529357 PMC3356611

[B23] Bystritsky A. Craske M. Maidenberg E. Vapnik T. Shapiro D. (2000). Autonomic reactivity of panic patients during a CO_2_ inhalation procedure. Depress. Anxiety 11 (1), 15–26. 10.1002/(sici)1520-6394(2000)11:1<15:aid-da3>3.0.co;2-w 10723631

[B24] Cahill L. (2010). “Chapter 3 - sex influences on brain and emotional memory: The burden of proof has shifted,” in Progress in brain research. Editor Ivanka S. (Netherlands: Elsevier). 10.1016/B978-0-444-53630-3.00003-8 21094884

[B25] Carrive P. Kuwaki T. (2017). “Orexin and central modulation of cardiovascular and respiratory function,” in Current topics in behavioral neurosciences. Editors Lawrence A. J. de Lecea L. (Netherlands: Elsevier). 10.1007/7854_2016_46 27909989

[B26] Charil A. Laplante D. P. Vaillancourt C. King S. (2010). Prenatal stress and brain development. Brain Res. Rev. 65 (1), 56–79. 10.1016/j.brainresrev.2010.06.002 20550950

[B27] Chung C. K. Remington N. D. Suh B. Y. (1995). Estrogen replacement therapy may reduce panic symptoms. J. Clin. Psychiatry 56 (11), 533. 7592508

[B28] Ciriello J. Caverson M. M. McMurray J. C. Bruckschwaiger E. B. (2013). Co-localization of hypocretin-1 and leucine-enkephalin in hypothalamic neurons projecting to the nucleus of the solitary tract and their effect on arterial pressure. Neuroscience 250, 599–613. 10.1016/j.neuroscience.2013.07.054 23912034

[B29] Cittaro D. Lampis V. Luchetti A. Coccurello R. Guffanti A. Felsani A. (2016). Histone modifications in a mouse model of early adversities and panic disorder: Role for Asic1 and neurodevelopmental genes. Sci. Rep. 6 (1), 25131. 10.1038/srep25131 27121911 PMC4848503

[B30] D'Amato F. R. Zanettini C. Lampis V. Coccurello R. Pascucci T. Ventura R. (2011). Unstable maternal environment, separation anxiety, and heightened CO_2_ sensitivity induced by gene-by-environment interplay. PLoS ONE 6 (4), e18637. 10.1371/journal.pone.0018637 21494633 PMC3072999

[B31] Díaz-Casares A. López-González M. V. Peinado-Aragonés C. A. Lara J. P. González-Barón S. Dawid-Milner M. S. (2009). Role of the parabrachial complex in the cardiorespiratory response evoked from hypothalamic defense area stimulation in the anesthetized rat. Brain Res. 1279, 58–70. 10.1016/j.brainres.2009.02.085 19376096

[B32] DiMicco J. A. Samuels B. C. Zaretskaia M. V. Zaretsky D. V. (2002). The dorsomedial hypothalamus and the response to stress: Part renaissance, part revolution. Pharmacol. Biochem. Behav. 71 (3), 469–480. 10.1016/s0091-3057(01)00689-x 11830181

[B33] Dlouhy B. J. Gehlbach B. K. Kreple C. J. Kawasaki H. Oya H. Buzza C. (2015). Breathing inhibited when seizures spread to the amygdala and upon amygdala stimulation. J. Neurosci. 35 (28), 10281–10289. 10.1523/jneurosci.0888-15.2015 26180203 PMC4502266

[B34] Donner N. Lowry C. (2013). Sex differences in anxiety and emotional behavior. Pflügers Archiv - Eur. J. Physiology 465 (5), 601–626. 10.1007/s00424-013-1271-7 23588380 PMC3805826

[B35] Drury A. N. (1918). The percentage of carbon dioxide in the alveolar air, and the tolerance to accumulating carbon dioxide in case of so-called "irritable heart. Heart 7, 165–173.

[B36] Dumont F. S. Biancardi V. Kinkead R. (2011). Hypercapnic ventilatory response of anesthetized female rats subjected to neonatal maternal separation: Insight into the origins of panic attacks? Respir. Physiology Neurobiol. 175 (2), 288–295. 10.1016/j.resp.2010.12.004 21147276

[B37] Dumont F. S. Kinkead R. (2011). Neonatal stress and abnormal hypercapnic ventilatory response of adult male rats: The role of central chemodetection and pulmonary stretch receptors. Respir. Physiology Neurobiol. 179 (2-3), 158–166. 10.1016/j.resp.2011.07.012 21824531

[B38] Dumont F. S. Kinkead R. (2010). Neonatal stress and attenuation of the hypercapnic ventilatory response in adult male rats: The role of carotid chemoreceptors and baroreceptors. Am. J. Physiol. Regul. Integr. Comp. Physiol. 299 (5), R1279–R1289. 10.1152/ajpregu.00446.2010 20811006

[B39] Edelmann M. N. Auger A. P. (2011). Epigenetic impact of simulated maternal grooming on estrogen receptor alpha within the developing amygdala. Brain, Behav. Immun. 25 (7), 1299–1304. 10.1016/j.bbi.2011.02.009 21352906 PMC3399737

[B40] Elliot-Portal E. Arias-Reyes C. Laouafa S. Tam R. Kinkead R. Soliz J. (2021). Cerebral erythropoietin prevents sex-dependent disruption of respiratory control induced by early life stress. Front. Physiology 12 (2280), 701344. 10.3389/fphys.2021.701344 PMC8720854 34987412

[B41] Estacio M. A. C. Yamada S. Tsukamura H. Hirunagi K. Maeda K.-i. (1996). Effect of fasting and immobilization stress on estrogen receptor immunoreactivity in the brain in ovariectomized female rats. Brain Res. 717 (1), 55–61. 10.1016/0006-8993(96)00022-4 8738253

[B42] Evrard H. C. Balthazart J. (2004). Rapid regulation of pain by estrogens synthesized in spinal dorsal horn neurons. J. Neurosci. 24 (33), 7225–7229. 10.1523/JNEUROSCI.1638-04.2004 15317848 PMC6729773

[B43] Feinstein J. S. Buzza C. Hurlemann R. Follmer R. L. Dahdaleh N. S. Coryell W. H. (2013). Fear and panic in humans with bilateral amygdala damage. Nat. Neurosci. 16 (3), 270–272. 10.1038/nn.3323 23377128 PMC3739474

[B44] Feinstein J. S. Gould D. Khalsa S. S. (2022). Amygdala-driven apnea and the chemoreceptive origin of anxiety. Biol. Psychol. 170, 108305. 10.1016/j.biopsycho.2022.108305 35271957 PMC10227885

[B45] Feng P. Vurbic D. Wu Z. Strohl K. P. (2007). Brain orexins and wake regulation in rats exposed to maternal deprivation. Brain Res. 1154 (0), 163–172. 10.1016/j.brainres.2007.03.077 17466285

[B46] Frye C. A. Koonce C. J. Edinger K. L. Osborne D. M. Walf A. A. (2008). Androgens with activity at estrogen receptor beta have anxiolytic and cognitive-enhancing effects in male rats and mice. Hormones Behav. 54 (5), 726–734. 10.1016/j.yhbeh.2008.07.013 PMC3623974 18775724

[B47] Fumagalli F. Molteni R. Racagni G. Riva M. A. (2007). Stress during development: Impact on neuroplasticity and relevance to psychopathology. Prog. Neurobiol. 81 (4), 197–217. 10.1016/j.pneurobio.2007.01.002 17350153

[B48] Garbarino S. Bardwell W. A. Guglielmi O. Chiorri C. Bonanni E. Magnavita N. (2020). Association of anxiety and depression in obstructive sleep apnea patients: A systematic review and meta-analysis. Behav. Sleep. Med. 18 (1), 35–57. 10.1080/15402002.2018.1545649 30453780

[B49] Garcia-Segura L. M. Azcoitia I. DonCarlos L. L. (2001). Neuroprotection by estradiol. Prog. Neurobiol. 63 (1), 29–60. 10.1016/S0301-0082(00)00025-3 11040417

[B50] Gardner W. N. (1996). The pathophysiology of hyperventilation disorders. Chest 109 (2), 516–534. 10.1378/chest.109.2.516 8620731

[B51] Gargaglioni L. H. Marques D. A. Patrone L. G. A. (2019). Sex differences in breathing. Comp. Biochem. Physiology Part A Mol. Integr. Physiology 238, 110543. 10.1016/j.cbpa.2019.110543 31445081

[B52] Genest S. E. Gulemetova R. Laforest S. Drolet G. Kinkead R. (2007). Neonatal maternal separation induces sex-specific augmentation of the hypercapnic ventilatory response in awake rat. J. Appl. Physiol. 102, 1416–1421. 10.1152/japplphysiol.00454.2006 17185497

[B53] Gerrits M. Grootkarijn A. Bekkering B. F. Bruinsma M. Boer J. A. D. Horst G. J. T. (2005). Cyclic estradiol replacement attenuates stress-induced c-Fos expression in the PVN of ovariectomized rats. Brain Res. Bull. 67 (1–2), 147–155. 10.1016/j.brainresbull.2005.06.021 16140174

[B54] Gestreau C. Bevengut M. Dutschmann M. (2008). The dual role of the orexin/hypocretin system in modulating wakefulness and respiratory drive. Curr. Opin. Pulm. Med. 14 (6), 512–518. 10.1097/MCP.0b013e32831311d3 18812827

[B55] Goldstein J. M. Jerram M. Abbs B. Whitfield-Gabrieli S. Makris N. (2010). Sex differences in stress response circuitry activation dependent on female hormonal cycle. J. Neurosci. 30 (2), 431–438. 10.1523/jneurosci.3021-09.2010 20071507 PMC2827936

[B56] Gorman J. M. Kent J. Martinez J. Browne S. Coplan J. Papp L. A. (2001). Physiological changes during carbon dioxide inhalation in patients with panic disorder, major depression, and premenstrual dysphoric disorder: Evidence for a central fear mechanism. Arch. Gen. Psychiatry 58 (2), 125–131. 10.1001/archpsyc.58.2.125 11177114

[B57] Gorman J. M. Kent J. M. Sullivan G. M. Coplan J. D. (2000). Neuroanatomical hypothesis of panic disorder, revised. Am. J. Psychiatry 157 (4), 493–505. 10.1176/appi.ajp.157.4.493 10739407

[B58] Gottschalk M. G. Richter J. Ziegler C. Schiele M. A. Mann J. Geiger M. J. (2019). Orexin in the anxiety spectrum: Association of a HCRTR1 polymorphism with panic disorder/agoraphobia, CBT treatment response and fear-related intermediate phenotypes. Transl. Psychiatry 9 (1), 75. 10.1038/s41398-019-0415-8 30718541 PMC6361931

[B59] Grafe L. A. Bhatnagar S. (2018). The contribution of orexins to sex differences in the stress response. Brain Res. 1731, 145893. 10.1016/j.brainres.2018.07.026 30081036 PMC6360123

[B60] Graham Y. P. Heim C. Goodman S. H. Miller A. H. Nemeroff C. B. (1999). The effects of neonatal stress on brain development: Implications for psychopathology. Dev. Psychopathol. 11 (3), 545–565. 10.1017/s0954579499002205 10532624

[B61] Grassi M. Caldirola D. Vanni G. Guerriero G. Piccinni M. Valchera A. (2013). Baseline respiratory parameters in panic disorder: A meta-analysis. J. Affect. Disord. 146 (2), 158–173. 10.1016/j.jad.2012.08.034 23107756

[B62] Gunnar M. R. (2003). Integrating neuroscience and psychological approaches in the study of early experiences. Ann. N. Y. Acad. Sci. 1008, 238–247. 10.1196/annals.1301.024 14998888

[B63] Guyenet P. G. Bayliss D. A. (2015). Neural control of breathing and CO_2_ homeostasis. Neuron 87 (5), 946–961. 10.1016/j.neuron.2015.08.001 26335642 PMC4559867

[B64] Guyenet P. G. Bayliss D. A. (2022). Central respiratory chemoreception. Handb. Clin. Neurol. 188, 37–72. 10.1016/b978-0-323-91534-2.00007-2 35965033 PMC10557475

[B65] Guyenet P. G. Stornetta R. L. Souza G. M. P. R. Abbott S. B. G. Shi Y. Bayliss D. A. (2019). The retrotrapezoid nucleus: Central chemoreceptor and regulator of breathing automaticity. Trends Neurosci. 42 (11), 807–824. 10.1016/j.tins.2019.09.002 31635852 PMC6825900

[B66] Hara Y. Waters E. M. McEwen B. S. Morrison J. H. (2015). Estrogen effects on cognitive and synaptic health over the lifecourse. Physiol. Rev. 95 (3), 785–807. 10.1152/physrev.00036.2014 26109339 PMC4491541

[B67] Harper R. M. Frysinger R. C. Trelease R. B. Marks J. D. (1984). State-dependent alteration of respiratory cycle timing by stimulation of the central nucleus of the amygdala. Brain Res. 306 (1–2), 1–8. 10.1016/0006-8993(84)90350-0 6466967

[B68] Harris G. C. Aston-Jones G. (2006). Arousal and reward: A dichotomy in orexin function. Trends Neurosci. 29 (10), 571–577. 10.1016/j.tins.2006.08.002 16904760

[B69] Hayward C. Killen J. D. Hammer L. D. Litt I. F. Wilson D. M. Simmonds B. (1992). Pubertal stage and panic attack history in sixth- and seventh-grade girls. Am. J. Psychiatry 149 (9), 1239–1243. 10.1176/ajp.149.9.1239 1503139

[B70] Heck A. L. Handa R. J. (2019). Sex differences in the hypothalamic–pituitary–adrenal axis’ response to stress: An important role for gonadal hormones. Neuropsychopharmacology 44 (1), 45–58. 10.1038/s41386-018-0167-9 30111811 PMC6235871

[B71] Hoppe L. J. Ipser J. Gorman J. M. Stein D. J. (2012). “Panic disorder,” in Handbook of clinical neurology. Editors MichaelAminoff F. B. J. Dick F. S. (Netherlands: Elsevier). 10.1016/B978-0-444-52002-9.00020-6 22608631

[B72] Iturriaga R. Alcayaga J. Chapleau M. W. Somers V. K. (2021). Carotid body chemoreceptors: Physiology, pathology, and implications for health and disease. Physiol. Rev. 101 (3), 1177–1235. 10.1152/physrev.00039.2019 33570461 PMC8526340

[B73] Johnson P. L. Truitt W. Fitz S. D. Minick P. E. Dietrich A. Sanghani S. (2010). A key role for orexin in panic anxiety. Nat. Med. 16 (1), 111–115. 10.1038/nm.2075 20037593 PMC2832844

[B74] Katzman M. A. Struzik L. Vijay N. Coonerty-Femiano A. Mahamed S. Duffin J. (2002). Central and peripheral chemoreflexes in panic disorder. Psychiatry Res. 113 (1-2), 181–192. 10.1016/s0165-1781(02)00238-x 12467957

[B75] Kaur S. De Luca R. Khanday M. A. Bandaru S. S. Thomas R. C. Broadhurst R. Y. (2020). Role of serotonergic dorsal raphe neurons in hypercapnia-induced arousals. Nat. Commun. 11 (1), 2769. 10.1038/s41467-020-16518-9 32488015 PMC7265411

[B76] Kaur S. Saper C. B. (2019). Neural circuitry underlying waking up to hypercapnia. Front. Neurosci. 13 (401), 401. 10.3389/fnins.2019.00401 31080401 PMC6497806

[B77] Kaur S. Wang J. L. Ferrari L. Thankachan S. Kroeger D. Venner A. (2017). A genetically defined circuit for arousal from sleep during hypercapnia. Neuron 96 (5), 1153–1167. 10.1016/j.neuron.2017.10.009 29103805 PMC5720904

[B78] Keshavarzi S. Sullivan R. K. P. Ianno D. J. Sah P. (2014). Functional properties and projections of neurons in the medial amygdala. J. Neurosci. 34 (26), 8699–8715. 10.1523/jneurosci.1176-14.2014 24966371 PMC6608208

[B79] Kinkead R. Gagnon M. Carrier M.-C. Fournier S. Ambrozio-Marques D. (2022). Amygdala-driven apnea: A breath of fresh air in respiratory neurobiology. Biol. Psychol. 170, 108307. 10.1016/j.biopsycho.2022.108307 35278529

[B80] Kinkead R. Montandon G. Bairam A. Lajeunesse Y. Horner R. L. (2009). Neonatal maternal separation disrupts regulation of sleep and breathing in adult male rats. Sleep 32 (12), 1611–1620. 10.1093/sleep/32.12.1611 20041597 PMC2786045

[B81] Kinkead R. Tenorio L. Drolet G. Bretzner F. Gargaglioni L. (2014). Respiratory manifestations of panic disorder in animals and humans: A unique opportunity to understand how supramedullary structures regulate breathing. Respir. Physiol. Neurobiol. 204 (0), 3–13. 10.1016/j.resp.2014.06.013 25038523

[B82] Klein D. F. (1993). False suffocation alarms, spontaneous panics, and related conditions. An integrative hypothesis. Archives General Psychiatry 50 (4), 306–317. 10.1001/archpsyc.1993.01820160076009 8466392

[B83] Koo J. W. Wohleb E. S. (2021). How stress shapes neuroimmune function: Implications for the neurobiology of psychiatric disorders. Biol. Psychiatry 90 (2), 74–84. 10.1016/j.biopsych.2020.11.007 33485589 PMC8126571

[B84] Kunte H. Harms L. Plag J. Hellweg R. Kronenberg G. (2014). Acute onset of panic attacks after transdermal estrogen replacement. General Hosp. Psychiatry 36 (5), e7. 10.1016/j.genhosppsych.2014.05.009 24973910

[B85] Li A. Nattie E. E. (2014). Orexin, cardio-respiratory function and hypertension. Front. Neurosci. 8, 22. 10.3389/fnins.2014.00022 24574958 PMC3921571

[B86] Lindheim S. R. Legro R. S. Bernstein L. Stanczyk F. Z. Vijod M. A. Presser S. C. (1992). Behavioral stress responses in premenopausal and postmenopausal women and the effects of estrogen. Am. J. Obstetrics Gynecol. 167 (6), 1831–1836. 10.1016/0002-9378(92)91783-7 1471706

[B87] Liu F. Day M. Muñiz L. C. Bitran D. Arias R. Revilla-Sanchez R. (2008). Activation of estrogen receptor-β regulates hippocampal synaptic plasticity and improves memory. Nat. Neurosci. 11 (3), 334–343. 10.1038/nn2057 18297067

[B88] Lovick T. A. (2014). Sex determinants of experimental panic attacks. Neurosci. Biobehav Rev. 46P3, 465–471. 10.1016/j.neubiorev.2014.03.006 24704571

[B89] Luchetti A. Oddi D. Lampis V. Centofante E. Felsani A. Battaglia M. (2015). Early handling and repeated cross-fostering have opposite effect on mouse emotionality. Front. Behav. Neurosci. 9 (93), 93. 10.3389/fnbeh.2015.00093 25954170 PMC4404916

[B90] Marcus J. N. Aschkenasi C. J. Lee C. E. Chemelli R. M. Saper C. B. Yanagisawa M. (2001). Differential expression of orexin receptors 1 and 2 in the rat brain. J. Comp. Neurology 435 (1), 6–25. 10.1002/cne.1190 11370008

[B91] Marek R. Strobel C. Bredy T. W. Sah P. (2013). The amygdala and medial prefrontal cortex: Partners in the fear circuit. J. Physiology 591 (10), 2381–2391. 10.1113/jphysiol.2012.248575 PMC3678031 23420655

[B92] Marques D. A. Carvalho D. d. Silva G. S. F. d. Szawka R. E. Anselmo-Franci J. A. Bícego K. C. (2015). Ventilatory, metabolic, and thermal responses to hypercapnia in female rats: Effects of estrous cycle, ovariectomy, and hormonal replacement. J. Appl. Physiology 119 (1), 61–68. 10.1152/japplphysiol.00254.2015 25930026

[B93] Meng X. D'Arcy C. (2012). Common and unique risk factors and comorbidity for 12-month mood and anxiety disorders among Canadians. Can. J. Psychiatry 57 (8), 479–487. 10.1177/070674371205700806 22854030

[B94] Moreira F. Gobira P. Viana T. Vicente M. Zangrossi H. Graeff F. (2013). Modeling panic disorder in rodents. Cell Tissue Res. 354, 119–125. 10.1007/s00441-013-1610-1 23584609

[B95] Nakamura A. Zhang W. Yanagisawa M. Fukuda Y. Kuwaki T. (2007). Vigilance state-dependent attenuation of hypercapnic chemoreflex and exaggerated sleep apnea in orexin knockout mice. J. Appl. Physiology 102 (1), 241–248. 10.1152/japplphysiol.00679.2006 16959906

[B96] Nardi A. E. Freire R. C. Zin W. A. (2009). Panic disorder and control of breathing. Respir. Physiology Neurobiol. 167, 133–143. 10.1016/j.resp.2008.07.011 18707030

[B97] Nattie E. Li A. (2011). “Central chemoreceptors: Locations and functions,” in Comprehensive physiology (New Jersey: John Wiley & Sons, Inc). 10.1002/cphy.c100083 PMC4802370 23728974

[B98] Nattie E. Li A. (2012). “Chapter 4 - respiration and autonomic regulation and orexin,” in Progress in brain research. Editor Anantha S. (Netherlands: Elsevier). 10.1016/B978-0-444-59489-1.00004-5 PMC4405125 22813968

[B99] Nillni Y. I. Pineles S. L. Rohan K. J. Zvolensky M. J. Rasmusson A. M. (2017). The influence of the menstrual cycle on reactivity to a CO2 challenge among women with and without premenstrual symptoms. Cogn. Behav. Ther. 46 (3), 239–249. 10.1080/16506073.2016.1236286 27687294 PMC6598439

[B100] Nillni Y. I. Toufexis D. J. Rohan K. J. (2011). Anxiety sensitivity, the menstrual cycle, and panic disorder: A putative neuroendocrine and psychological interaction. Clin. Psychol. Rev. 31 (7), 1183–1191. 10.1016/j.cpr.2011.07.006 21855828 PMC3176921

[B101] Österlund M. K. (2010). Underlying mechanisms mediating the antidepressant effects of estrogens. Biochimica Biophysica Acta (BBA) - General Subj. 1800 (10), 1136–1144. 10.1016/j.bbagen.2009.11.001 19900508

[B102] Östlund H. Keller E. Hurd Y. L. (2003). Estrogen receptor gene expression in relation to neuropsychiatric disorders. Ann. N. Y. Acad. Sci. 1007 (1), 54–63. 10.1196/annals.1286.006 14993040

[B103] Petrov T. Krukoff T. L. Jhamandas J. H. (1995). Convergent influence of the central nucleus of the amygdala and the paraventricular hypothalamic nucleus upon brainstem autonomic neurons as revealed by c-fos expression and anatomical tracing. J. Neurosci. Res. 42 (6), 835–845. 10.1002/jnr.490420612 8847745

[B104] Pfaus J. G. Jones S. L. Flanagan-Cato L. M. Blaustein J. D. (2015). “Chapter 50 - female sexual behavior,” in Knobil and neill's physiology of reproduction. Editors Plant T. M. Zeleznik A. J. Fourth Edition (San Diego: Academic Press).

[B105] Pigott T. A. (2003). Anxiety disorders in women. Psychiatric Clin. N. Am. 26 (3), 621–672. vi-vii. 10.1016/s0193-953x(03)00040-6 14563101

[B106] Price W. A. Heil D. (1988). Estrogen-induced panic attacks. Psychosomatics 29 (4), 433–435. 10.1016/s0033-3182(88)72347-6 3227101

[B107] Putnam R. W. Conrad S. C. Gdovin M. J. Erlichman J. S. Leiter J. C. (2005). Neonatal maturation of the hypercapnic ventilatory response and central neural CO_2_ chemosensitivity. Respir. Physiol. Neurobiol. 149, 165–179. 10.1016/j.resp.2005.03.004 15876557 PMC1255969

[B108] Rappaport L. M. Sheerin C. Carney D. M. Towbin K. E. Leibenluft E. Pine D. S. (2017). Clinical correlates of carbon dioxide hypersensitivity in children. J. Am. Acad. Child Adolesc. Psychiatry 56 (12), 1089–1096. 10.1016/j.jaac.2017.09.423 29173743 PMC5762134

[B109] Reardon L. E. Leen-Feldner E. W. Hayward C. (2009). A critical review of the empirical literature on the relation between anxiety and puberty. Clin. Psychol. Rev. 29 (1), 1–23. 10.1016/j.cpr.2008.09.005 19019513 PMC2652567

[B110] Reed V. Wittchen H. U. (1998). DSM-IV panic attacks and panic disorder in a community sample of adolescents and young adults: How specific are panic attacks? J. Psychiatr. Res. 32 (6), 335–345. 10.1016/s0022-3956(98)00014-4 9844949

[B111] Rhone A. E. Kovach C. K. Harmata G. I. S. Sullivan A. W. Tranel D. Ciliberto M. A. (2020). A human amygdala site that inhibits respiration and elicits apnea in pediatric epilepsy. JCI Insight 5 (6), e134852. 10.1172/jci.insight.134852 32163374 PMC7213805

[B112] Ritchie E. C. (1992). Treatment of gas mask phobia. Mil. Med. 157 (2), 104–106. 10.1093/milmed/157.2.104 1603385

[B113] Roberson-Nay R. Kendler K. S. (2011). Panic disorder and its subtypes: A comprehensive analysis of panic symptom heterogeneity using epidemiological and treatment seeking samples. Psychol. Med. 41, 2411–2421. 10.1017/S0033291711000547 21557895 PMC4096693

[B114] Roberson-Nay R. Klein D. F. Klein R. G. Mannuzza S. Moulton J. L. Guardino M. (2010). Carbon dioxide hypersensitivity in separation-anxious offspring of parents with panic disorder. Biol. psychiatry 67 (12), 1171–1177. 10.1016/j.biopsych.2009.12.014 20172505 PMC3617557

[B115] Rodrigues S. M. LeDoux J. E. Sapolsky R. M. (2009). The influence of stress hormones on fear circuitry. Annu. Rev. Neurosci. 32 (1), 289–313. 10.1146/annurev.neuro.051508.135620 19400714

[B116] Rosin D. L. Chang D. A. Guyenet P. G. (2006). Afferent and efferent connections of the rat retrotrapezoid nucleus. J. Comp. Neurology 499 (1), 64–89. 10.1002/cne.21105 16958085

[B117] Schenberg L. (2016). “A neural systems approach to the study of respiratory-type panic disorder,” in Panic disorder. Editors Nardi A. RCR R. (Switzerland: Springer international Publishing).

[B118] Schimitel F. G. de Almeida G. M. Pitol D. N. Armini R. S. Tufik S. Schenberg L. C. (2012). Evidence of a suffocation alarm system within the periaqueductal gray matter of the rat. Neuroscience 200, 59–73. 10.1016/j.neuroscience.2011.10.032 22062132

[B119] Shonkoff J. P. Boyce W. T. McEwen B. S. (2009). Neuroscience, molecular biology, and the childhood roots of health disparities: Building a new framework for health promotion and disease prevention. JAMA 301 (21), 2252–2259. 10.1001/jama.2009.754 19491187

[B120] Shors T. J. Chua C. Falduto J. (2001). Sex differences and opposite effects of stress on dendritic spine density in the male versus female Hippocampus. J. Neurosci. 21 (16), 6292–6297. 10.1523/jneurosci.21-16-06292.2001 11487652 PMC6763131

[B121] Shughrue P. J. Lane M. V. Merchenthaler I. (1997). Comparative distribution of estrogen receptor-alpha and -beta mRNA in the rat central nervous system. J. Comp. Neurol. 388 (4), 507–525. 10.1002/(sici)1096-9861(19971201)388:4<507:aid-cne1>3.0.co;2-6 9388012

[B122] Silveyra P. Cataldi N. I. Lux-Lantos V. Libertun C. (2009). Gonadal steroids modulated hypocretin/orexin type-1 receptor expression in a brain region, sex and daytime specific manner. Regul. Pept. 158 (1–3), 121–126. 10.1016/j.regpep.2009.08.002 19699765

[B123] Simerly R. B. Young B. J. (1991). Regulation of estrogen receptor messenger ribonucleic acid in rat hypothalamus by sex steroid hormones. Mol. Endocrinol. 5 (3), 424–432. 10.1210/mend-5-3-424 1890991

[B124] Sinha S. Papp L. A. Gorman J. M. (2000). How study of respiratory physiology aided our understanding of abnormal brain function in panic disorder. J. Affect Disord. 61 (3), 191–200. 10.1016/s0165-0327(00)00337-2 11163421

[B125] Smith H. R. Leibold N. K. Rappoport D. A. Ginapp C. M. Purnell B. S. Bode N. M. (2018). Dorsal raphe serotonin neurons mediate CO_2_-induced arousal from sleep. J. Neurosci. 38 (8), 1915–1925. 10.1523/jneurosci.2182-17.2018 29378860 PMC5824737

[B126] Smoller J. W. Gallagher P. J. Duncan L. E. McGrath L. M. Haddad S. A. Holmes A. J. (2014). The human ortholog of acid-sensing ion channel gene ASIC1a is associated with panic disorder and amygdala structure and function. Biol. Psychiatry 76 (11), 902–910. 10.1016/j.biopsych.2013.12.018 24529281 PMC4103972

[B127] Soliz J. Tam R. Kinkead R. (2016). Neonatal maternal separation augments carotid body response to hypoxia in adult males but not female rats. Front. Physiology 7 (432), 432. 10.3389/fphys.2016.00432 PMC5037225 27729873

[B128] Song N. Guan R. Jiang Q. Hassanzadeh C. J. Chu Y. Zhao X. (2016). Acid-sensing ion channels are expressed in the ventrolateral medulla and contribute to central chemoreception. Sci. Rep. 6 (1), 38777. 10.1038/srep38777 27934921 PMC5146928

[B129] Stein M. B. Millar T. W. Larsen D. K. Kryger M. H. (1995). Irregular breathing during sleep in patients with panic disorder. Am. J. Psychiatry 152 (8), 1168–1173. 10.1176/ajp.152.8.1168 7625465

[B130] Sunanaga J. Deng B.-S. Zhang W. Kanmura Y. Kuwaki T. (2009). CO2 activates orexin-containing neurons in mice. Respir. Physiology Neurobiol. 166 (3), 184–186. 10.1016/j.resp.2009.03.006 19442935

[B131] Tan X.-j. Dai Y.-b. Wu W.-f. Kim H.-J. Barros R. P. A. Richardson T. I. (2012). Reduction of dendritic spines and elevation of GABAergic signaling in the brains of mice treated with an estrogen receptor β ligand. Proc. Natl. Acad. Sci. 109 (5), 1708–1712. 10.1073/pnas.1121162109 22307635 PMC3277134

[B132] Taugher R. J. Dlouhy B. J. Kreple C. J. Ghobbeh A. Conlon M. M. Wang Y. (2020). The amygdala differentially regulates defensive behaviors evoked by CO_2_ . Behav. Brain Res. 377, 112236. 10.1016/j.bbr.2019.112236 31536735 PMC6829583

[B133] Tenorio-Lopes L. Fournier S. Henry M. S. Bretzner F. Kinkead R. (2020). Disruption of estradiol regulation of orexin neurons: A novel mechanism in excessive ventilatory response to CO2 inhalation in a female rat model of panic disorder. Transl. Psychiatry 10 (1), 394. 10.1038/s41398-020-01076-x 33173029 PMC7656265

[B134] Tenorio-Lopes L. Henry M. S. Marques D. Tremblay M. È. Drolet G. Bretzner F. (2017). Neonatal maternal separation opposes the facilitatory effect of castration on the respiratory response to hypercapnia of the adult male rat: Evidence for the involvement of the medial amygdala. J. Neuroendocrinol. 29 (12), e12550. 10.1111/jne.12550 29063642

[B135] Tenorio-Lopes L. Kinkead R. (2021). Sex-specific effects of stress on respiratory control: Plasticity, adaptation, and dysfunction. Compr. Physiol. 11, 2097–2134. 10.1002/cphy.c200022 34107062

[B136] Tian Z. Wang Y. Zhang N. Guo Y.-y. Feng B. Liu S.-b. (2013). Estrogen receptor GPR30 exerts anxiolytic effects by maintaining the balance between GABAergic and glutamatergic transmission in the basolateral amygdala of ovariectomized mice after stress. Psychoneuroendocrinology 38 (10), 2218–2233. 10.1016/j.psyneuen.2013.04.011 23669322

[B137] Ulrich-Lai Y. M. Herman J. P. (2009). Neural regulation of endocrine and autonomic stress responses. Nat. Rev. Neurosci. 10 (6), 397–409. 10.1038/nrn2647 19469025 PMC4240627

[B138] van Duinen M. A. Schruers K. R. J. Maes M. Griez E. J. L. (2007). CO2 challenge induced HPA axis activation in panic. Int. J. Neuropsychopharmacol. 10 (06), 797–804. 10.1017/S1461145706007358 17076937

[B139] Veening J. G. Swanson L. W. Sawchenko P. E. (1984). The organization of projections from the central nucleus of the amygdala to brainstem sites involved in central autonomic regulation: A combined retrograde transport-immunohistochemical study. Brain Res. 303 (2), 337–357. 10.1016/0006-8993(84)91220-4 6204716

[B140] Venkatraman A. Edlow B. L. Immordino-Yang M. H. (2017). The brainstem in emotion: A review. Front. Neuroanat. 11 (15), 15. 10.3389/fnana.2017.00015 28337130 PMC5343067

[B141] Viau V. Meaney M. J. (1991). Variations in the hypothalamic-pituitary-adrenal response to stress during the estrous cycle in the rat. Endocrinology 129 (5), 2503–2511. 10.1210/endo-129-5-2503 1657578

[B142] Vollmer L. L. Ghosal S. McGuire J. L. Ahlbrand R. L. Li K.-Y. Santin J. M. (2016). Microglial acid sensing regulates carbon dioxide-evoked fear. Biol. Psychiatry 80 (7), 541–551. 10.1016/j.biopsych.2016.04.022 27422366 PMC5014599

[B143] Walf A. A. Frye C. A. (2006). A review and update of mechanisms of estrogen in the hippocampus and amygdala for anxiety and depression behavior. Neuropsychopharmacology 31 (6), 1097–1111. 10.1038/sj.npp.1301067 16554740 PMC3624621

[B144] Wang X. Guan R. Zhao X. Chen J. Zhu D. Shen L. (2021). TASK1 and TASK3 in orexin neuron of lateral hypothalamus contribute to respiratory chemoreflex by projecting to nucleus tractus solitarius. FASEB J. 35 (5), e21532. 10.1096/fj.202002189R 33817828

[B145] Wilhelm F. H. Gevirtz R. Roth W. T. (2001). Respiratory dysregulation in anxiety, functional cardiac, and pain disorders. Assessment, phenomenology, and treatment. Behav. Modif. 25 (4), 513–545. 10.1177/0145445501254003 11530714

[B146] Wilhelm F. H. Roth W. T. (2001). The somatic symptom paradox in DSM-IV anxiety disorders: Suggestions for a clinical focus in psychophysiology. Biol. Psychol. 57 (1–3), 105–140. 10.1016/S0301-0511(01)00091-6 11454436

[B147] Williams R. H. Jensen L. T. Verkhratsky A. Fugger L. Burdakov D. (2007). Control of hypothalamic orexin neurons by acid and CO2. PNAS 104 (25), 10685–10690. 10.1073/pnas.0702676104 17563364 PMC1965573

[B148] Woolley C. McEwen B. (1992). Estradiol mediates fluctuation in hippocampal synapse density during the estrous cycle in the adult rat. J. Neurosci. 12 (7), 2549–2554. 10.1523/jneurosci.12-07-02549.1992 1613547 PMC6575846

[B149] Woolley C. S. (2007). Acute effects of estrogen on neuronal physiology. Annu. Rev. Pharmacol. Toxicol. 47 (1), 657–680. 10.1146/annurev.pharmtox.47.120505.105219 16918306

[B150] World-Health-Organization (1992). The ICD-10 classification of mental and behavioural disorders: Clinical descriptions and diagnostic guidelines. Genève: World-Health-Organization.

[B151] Wu J. Xu H. Shen W. Jiang C. (2004). Expression and coexpression of CO2-sensitive Kir channels in brainstem neurons of rats. J. Membr. Biol. 197 (3), 179–191. 10.1007/s00232-004-0652-4 15042349

[B152] Yang C. F. Kim E. J. Callaway E. M. Feldman J. L. (2020). Monosynaptic projections to excitatory and inhibitory preBötzinger complex neurons. Front. Neuroanat. 14 (58), 58. 10.3389/fnana.2020.00058 33013329 PMC7507425

[B153] Zhang W.-H. Zhang J.-Y. Holmes A. Pan B.-X. (2021). Amygdala circuit substrates for stress adaptation and adversity. Biol. Psychiatry 89 (9), 847–856. 10.1016/j.biopsych.2020.12.026 33691931

[B154] Ziemann A. E. Allen J. E. Dahdaleh N. S. Drebot I. I. Coryell M. W. Wunsch A. M. (2009). The amygdala is a chemosensor that detects carbon dioxide and acidosis to elicit fear behavior. Cell 139 (5), 1012–1021. 10.1016/j.cell.2009.10.029 19945383 PMC2808123

